# Type 1 interferon mediated signaling is indispensable for eliciting anti-tumor responses by *Mycobacterium indicus pranii*

**DOI:** 10.3389/fimmu.2023.1104711

**Published:** 2023-04-14

**Authors:** Gargi Roy, Anush Chakraborty, Bharati Swami, Lalit Pal, Charvi Ahuja, Soumen Basak, Sangeeta Bhaskar

**Affiliations:** ^1^ Product Development Cell, National Institute of Immunology, New Delhi, India; ^2^ Systems Immunology Lab, National Institute of Immunology, New Delhi, India

**Keywords:** *Mycobacterium indicus pranii*, tumor immunotherapy, type 1 interferon signaling, dendritic cells, regulatory T cells, CD8^+^ T cells

## Abstract

**Introduction:**

The evolving tumor secretes various immunosuppressive factors that reprogram the tumor microenvironment (TME) to become immunologically cold. Consequently, various immunosuppressive cells like Tregs are recruited into the TME which in turn subverts the anti-tumor response of dendritic cells and T cells.Tumor immunotherapy is a popular means to rejuvenate the immunologically cold TME into hot. *Mycobacterium indicus pranii* (MIP) has shown strong immunomodulatory activity in different animal and human tumor models and has been approved for treatment of lung cancer (NSCLC) patients as an adjunct therapy. Previously, MIP has shown TLR2/9 mediated activation of antigen presenting cells/Th1 cells and their enhanced infiltration in mouse melanoma but the underlying mechanism by which it is modulating these immune cells is not yet known.

**Results:**

This study reports for the first time that MIP immunotherapy involves type 1 interferon (IFN) signaling as one of the major signaling pathways to mediate the antitumor responses. Further, it was observed that MIP therapy significantly influenced frequency and activation of different subsets of T cells like regulatory T cells (Tregs) and CD8+ T cells in the TME. It reduces the migration of Tregs into the TME by suppressing the expression of CCL22, a Treg recruiting chemokine on DCs and this process is dependent on type 1 IFN. Simultaneously, in a type 1 IFN dependent pathway, it enhances the activation and effector function of the immunosuppressive tumor resident DCs which in turn effectively induce the proliferation and effector function of the CD8^+^ T cells.

**Conclusion:**

This study also provides evidence that MIP induced pro-inflammatory responses including induction of effector function of conventional dendritic cells and CD8^+^ T cells along with reduction of intratumoral Treg frequency are essentially mediated in a type 1 IFN-dependent pathway.

## 1 Introduction

The role of the immune system is crucial in protection against development of tumors as well as in response to different types of therapy. On recognition of tumor cells, the adaptive and innate arms of the immune system, specifically the Th1 branch, function in collaboration to generate a protective anti-tumor immune response. However, despite being recognized by immune cells, the majority of actively proliferating tumors are not effectively rejected due to negative regulatory mechanisms established by the growing tumor (1). As the cancer cells multiply, they secrete a variety of immunosuppressive factors that actively suppress the anti-tumor immune responses by dysregulating the effector function of the Th1 branch of the immune system (2, 3). Tumor immunotherapy plays a significant role in reversing such immunosuppressive tumor promoting conditions.

For tumor immunotherapy to be successful, it must accomplish two key goals: first, the suppression or reversal of the immunosuppressive regulatory mechanisms exerted by the proliferating tumor which leads to down-regulation of therapy-induced responses; second, induction of an immune response that can efficiently eliminate the cancerous cells (3, 4). A significant proportion of immunotherapy research studies focus on the activation of antitumor CD8^+^ T cells and γδ T cells which have excellent cytotoxic properties against tumor cells (5, 6). For effective elimination of tumor cells, APCs specifically DCs must cross-present tumor-associated antigens on their MHC class-I molecules. When exposed to danger or inflammatory signals, DCs get activated and stimulate cross-priming of CD8^+^ T cells to establish effective cytotoxic T lymphocyte (CTL) activity (7). Activated CD8^+^ T cells are capable of tumor cell killing either by inducing programmed cell death (apoptosis) through the Fas/Fas ligand pathway or by secreting perforin and Granzyme (8).

Regulatory T cells (Tregs) play a key role in maintaining peripheral tolerance and immune homeostasis. Their physiological function is to limit tissue damage by preventing excessive inflammation (8, 9). But in the TME, higher frequency of Tregs inhibits protective anti-tumor immune responses (10). There are numerous evidences which show that inhibition of Treg cell function or their infiltration in the TME significantly increases anti-tumor immune responses (11). However, detrimental autoimmunity is linked to systemic Treg depletion. So, targeting the Tregs present specifically in the TME is crucial for inducing proper anti-tumor response. For instance, terminally differentiated Tregs are the most common Treg cell type present in tumor tissues and they serve as excellent targets rather than the whole systemic Treg population (11). These cells have increased expression of PD-1, GITR, CTLA-4 and CCR4 in addition to other cell surface markers which inhibit anti-tumor responses (12). Specific monoclonal antibodies are available to target these proteins. Another efficient approach of tumor immunotherapy is to decrease the Treg-cells frequency/function along with increasing the activation and effector function of tumor-specific CD8^+^ T cells. For this, innate immune cells like dendritic cells (DCs) and macrophages play a crucial role in activation of T cells. Therefore, the interplay of innate and adaptive arms of the immune system is central to induce effective anti-tumor responses.

*Mycobacterium indicus pranii*, a non-pathogenic strain of *Mycobacterium* has demonstrated excellent anti-tumor activity in mouse model of tumor as well as in different clinical trials. Based on the encouraging results (13, 14), it is approved as an adjunct therapy for human use for non-small cell lung cancer patients by DCGI. In a murine tumor model, MIP treatment increases the infiltration and activation of CD4^+^, CD8^+^ T cells, NK and NKT cells as well as APCs resulting in secretion of Th1 type pro-inflammatory cytokines in TME. It is a potent TLR2 and TLR9 ligand and mediates anti-tumor immune responses in MyD88 dependent pathway. Another interesting observation in the mouse model of melanoma was significantly reduced frequency of regulatory T cells in MIP treated tumors as compared to control tumors (15–17). In two recent studies from our group, it has been observed that MIP nano-formulation activates the tumor residing immune cells which ultimately leads to the reduced tumor growth (18). Additionally, MIP suppresses the dissemination of tumor cells *in vivo*, by regulating the expression of MMP9 and CXCR4 on these cells (19). However, the detailed mechanism by which MIP immunotherapy modulates innate and adaptive immune system, particularly dendritic cells, Tregs and CD8^+^ T cells have not been studied in detail. In light of this, the present study demonstrates the mechanism by which tumor infiltrating dendritic cells respond to MIP immunotherapy to regulate CD8^+^ T cells and Tregs in the TME. We have shown that MIP is a potent inducer of IFN-α from mouse plasmacytoid DCs and mediates its anti-tumor activity through type 1 IFN dependent pathway. Importantly, type 1 IFN signaling plays a central role in MIP mediated immunomodulation of innate and adaptive immune cells. This study also provides evidence that MIP induced pro-inflammatory responses including induction of effector function of conventional dendritic cells and CD8^+^ T cells along with reduction of intratumoral Treg frequency are essentially mediated in a type 1 IFN-dependent pathway.

## 2 Materials and methods

### 2.1 Ethical approval

The study protocol was approved by the Animal Ethics Committee of the National Institute of Immunology (IAEC#558/20), (IBSC#403/20). Experimental procedures followed the guidelines of the Animal Ethics and Biosafety Committee of the National Institute of Immunology (New Delhi, India).

### 2.2 Animals

Inbred C57BL/6 mice and type 1 IFN receptor knockout (IFNR1^-/-^) mice were bred and housed in germ-free condition at the small animal facility of the National Institute of Immunology, New Delhi, India. IFNR1 heterozygous mating pairs were set up for generating IFNR1^-/-^ mice and genotyping was done for each litter.

### 2.3 Cell lines

B16F10 melanoma and Lewis lung carcinoma cell lines (obtained from American Type Culture Collection) were cultured in RPMI 1640 medium. Culture media was supplemented with 10% FBS (HIMEDIA) and 1% antibiotic-antimycotic solution and cells were grown in 37°C incubator with 5% CO2 and 95% humidified air. These cells were free of mycoplasma contamination.

### 2.4 Physical disruption of Mycobacterium indicus pranii

MIP culture was maintained on Lowenstein-Jensen medium (LJ) slants (BD Difco) and kept at -80°C. It was cultured in Middlebrook 7H9 medium (BD Difco) containing 0.2% glycerol, 0.05% Tween 80 and 10% albumin-dextrose-catalase enrichment (BD Difco) as a shake flask culture. Bacteria were harvested in the log growth phase by centrifugation at 7000xg for 15 minutes, washed twice with PBS, and suspended in chilled phosphate buffer saline containing 0.05% Polysorbate-20, 20mM EDTA and 1x Protease inhibitor cocktail (Sigma). This suspension of MIP was sonicated for 50 cycles (45 secs ON/15 secs OFF) at 60% power using an ultrasonicator. The entire procedure was done by placing the tube in ice in order to dissipate the heat generated by sonication. Sonicated cells were collected by centrifuging the cell suspension at 10,000xg for 10 minutes. It was then stored at -80° C and further used for immunization & treatment in the tumor bearing mice.

### 2.5 Tumor implantation

For tumor induction, 5 x 10^4^ B16F10 cells were subcutaneously injected in the right flank of syngeneic C57BL/6 mice. For the treatment regimen, 5 × 10^6^ fragmented and dead MIP/100 μl PBS was administered peritumorally on day 3 after tumor implantation. This was followed by 2 more weekly doses. Control animals were injected with 100 μl PBS using the same schedule. Tumor dimensions were measured with the help of Vernier caliper. Tumor volume was calculated using the formula V = 0.5 × L × W^2^, where V is tumor volume, L is longer dimension and W is the shorter dimension of tumor. Animals were euthanized by CO_2_ asphyxiation method on day 23-24 post tumor implantation, for further *ex-vivo* experiments

### 2.6 Preparation of tumor infiltrating mononuclear cells

Subcutaneously implanted tumors were removed from mice and single cell suspensions were prepared by enzymatic digestion. Resected tumors were weighed, mechanically disrupted, incubated with 1 mg/ml collagenase (Sigma) and 0.05 mg/mL DNAse I (Sigma) in a shaker incubator for 45 minutes at 37°C and filtered through a 40 μm cell strainer. Centrifuged at 400 g for 10 minutes. The pellet was further resuspended in 1x RBC lysis buffer (BD Pharm lyse) and kept at RT for 3 minutes to disrupt the RBCs. It was further washed with 1x PBS and centrifuged at 400 g for 10 minutes. Pellet was resuspended in incomplete RPMI. Cell suspension was layered over Ficoll-paque (Himedia) and centrifuged at 850 g for 40 minutes at 22°C with zero acceleration and brake. The buffy coat containing the tumor infiltrating leukocytes (TILs) at inter-phase was collected, cells were washed with 1x PBS and finally resuspended in FACS buffer (1x phosphate-buffered saline, pH 7.2, 1% fetal bovine serum and 1 mM EDTA) for flow cytometric analysis.

### 2.7 Flow cytometry

Single cell suspension of mononuclear cells obtained from tumor mass, were stained with antibodies against various cell surface and intracellular markers. Briefly, the antibody master mix was prepared in FACS buffer. The cell suspension was centrifuged for 5 minutes at 4°C. 100 µl of antibody master mix was added per tube containing 50-100 x 10^5^ cells. The tubes were incubated in dark for 30 minutes at 4°C. The centrifuge was pre-cooled to 4°C. The cells were washed with 1 ml of FACS buffer. The supernatant was removed and cell pellet was resuspended in FACS buffer.

For intracellular staining of cytokines, tumor infiltrating leukocytes were re-stimulated by incubation with PMA (10 ng/ml) ionomycin (750 ng/ml) and Golgi stop (BD Biosciences; 1 μl for 10^6^ cells/ml) for 5 hours at 37°C. PMA and ionomycin was purchased from Sigma. Next, stimulated cells were first stained for surface markers, fixed and permeabilized with Foxp3/Transcription Factor fixation buffer (Ebioscience) for 30 minutes in dark at 4°C and then cells were washed with 1X permeabilization buffer (Ebioscience). For intracellular cytokine staining, antibodies specific for mouse IFN-γ, IL-10, IL-12, IL-6 were diluted in 1x permeabilization buffer according to the recommended dilution and kept at 4°C in dark for 45 minutes. The cells were washed and resuspended in FACS buffer for further analysis. Flow cytometric analysis was done on BD FACS Verse or FACS Canto II flow cytometer and data were analyzed using FlowJo v2 software. All the analysis was performed using the antibodies listed in **Table S1**.

### 2.8 Adoptive transfer of total splenocytes

For adoptive transfer, single-cell suspensions from the spleen of healthy wild type mice were labeled with carboxyfluorescein diacetate succinimidyl ester (CFSE; Invitrogen) according to the manufacturer’s instructions. Briefly, a cell suspension of 1x10^6^ cells/ml was prepared in 1x PBS. These cells were further stained with CFSE for 20 minutes at 37°C using a 5 μM staining solution prepared in 1x PBS. After washing off the unbound dye, the CFSE stained splenocytes (3 x 10^7^ cells per recipient mouse) were injected intravenously through retro-orbital plexus into tumor-bearing recipient mice, 24 hours after administering the 3^rd^ dose of MIP.

### 2.9 RNA isolation and quantitative RT-PCR

Tumor tissues were mechanically digested followed by RBC lysis and density gradient centrifugation to enrich the tumor infiltrating leukocytes from the tumor tissue. Total RNA from TILs was extracted using RNA iso-plus obtained from TAKARA following the manufacturer’s recommendations. First strand complementary DNA (cDNA) was synthesized from total RNA using the PrimeScript First-Strand cDNA Synthesis Kit with PrimeScript Reverse Transcriptase according to the manufacturer’s protocol (TAKARA). Quantitative RT-PCR was performed with *SYBR Premix Ex Taq* (Tli RNase H Plus) by TAKARA following the manufacturer’s protocol. The expression levels of each transcript were normalized to the housekeeping gene GAPDH. The primer sequences used are enlisted in **Table S2**.

### 2.10 Bone marrow-derived dendritic cells preparation

Bone marrow from tibiae and femurs of C57Bl/6 wild type (6–8 weeks) mice were isolated and cultured in RPMI medium [containing 100 U/ml of penicillin and supplemented with 10% FBS and 20 ng/ml of mouse DC growth factor, GM-CSF] at 37°C and 5% of CO2 for 7 days. The media was replenished every 2 days. After 7 days, ~90% of cells were positive for CD11c (pan DC marker). They were regarded as the immature BMDCs.

### 2.11 Generation of tumor antigen pulsed BMDCs

B16F10 and LLC cells were grown and lysed by five freeze/thaw cycles in liquid nitrogen and in 37°C water-bath. Centrifugation done at ~10,000xg for 15 mins. Supernatant was filtered and protein concentration was determined by BCA. Immature BMDCs (1x10^6^) were pulsed *in vitro* with 100 μg/ml tumor lysate in 12-well flat-bottomed tissue culture plates for 60 hours to generate tumor antigen pulsed mature BMDCs.

### 2.12 Generation of tumor specific T cells *ex vivo*

CD3^+^ T cells were sorted from the spleen of advanced tumor bearing mice using the CD3^+^ T cell MACS kit (STEMCELL Technologies). 1 x 10^5^ T cells were co-cultured for 4-5 days in the presence of tumor antigen pulsed BMDCs isolated from healthy animals. They were cultured in RPMI medium containing 100 U/ml of penicillin and supplemented with 10% FBS, 2 mM L-glutamine and 2.5 μM β-mercaptoethanol.

### 2.13 Enrichment of naïve CD4^+^T cells, Tregs, pan DC and pDC

All the sub-populations of immune cells were enriched from respective tissues using MACS as per the manufacturer’s instructions. Enrichment kit for pDCs, Tregs, pan DCs and naïve CD4^+^ T cells were obtained from Miltenyi biotech. CD3^+^ T cell isolation was done using the CD3^+^ T cell isolation kit obtained from STEMCELL Technologies.

### 2.14 DC suppression assay

To generate tumor specific T cells, CD3^+^ T cells were obtained from spleens of advanced tumor-bearing animals using CD3^+^ T cell isolation kit (STEMCELL Technologies) and then added to cultures containing tumor antigen pulsed BMDCs (10:1). These were co-cultured for 4 days. Tumor specific CD3^+^ T cells were labeled with CFSE after MACS sorting. Next, tumor-pulsed BMDCs were added to the CFSE-labeled tumor-specific T cells (1:1) and co-cultured for 48 hours followed by addition of sorted non-pulsed CD11c^+^ DCs isolated from tumor draining lymph node (1:1:1 ratio) in a flat-bottom 96-well plate. CFSE dilution (readout for proliferation of T cells) was analyzed after 4 days.

### 2.15 Treg suppression assay

Naive CD4^+^CD25^-^ T cells (5 × 10^4^) isolated from the spleen of tumor bearing mice and labeled with CFSE. These were then cultured in flat-bottom 96 well plates with 5 x 10^4^ CD4^+^CD25^+^ T cells (90% FoxP3^+^) isolated from the tumor draining lymph nodes of control or MIP treated animals. To this, 10^4^ splenocytes of RAG^-/-^ mice (Antigen presenting cells) were added. The plates were pre-coated with 5 μg/ml anti-CD3 and 5 μg/ml anti-CD28 monoclonal antibody for 1 hour at 37°C for polyclonal stimulation. After 4 days of co-culture, proliferation of naïve T cells in presence of Tregs were analyzed in terms of CFSE dilution using flow cytometry.

### 2.16 Antigen presentation assay

CD3^+^ T cells were sorted from spleens of tumor bearing animals with advanced tumors (>30 days) using CD3^+^ T cell MACS kit (STEMCELL Technologies). These CD3^+^ T cells were cultured for 4-5 days with tumor pulsed BMDCs at a 10:1 ratio to generate tumor-specific T cells. Tumor-specific T cells were then CFSE labeled after MACS sorting and added to flat-bottom 96-well plates which contained sorted unpulsed DCs isolated from tumor (10:1 ratio). Cultures were analyzed by flow cytometry for CFSE dilution after 5 days.

### 2.17 Tumor specific cytokine production

Tumor draining lymph nodes from control and MIP treated animals were isolated. Single cell suspension was prepared and equal number of cells from control and MIP treated group were cultured in RPMI medium containing 100 U/ml of penicillin and supplemented with 10% FBS for 48-72 hours in the presence of B16 tumor lysate (100 μg/ml). The concentrations of tumor antigen specific cytokines like IFN-α (Elabscience), IL-12 (BD) and IL-6 (BD) in cell-free culture supernatant were quantified by ELISA according to the manufacturer’s instructions.

### 2.18 Estimation of chemokine secretion

Tumor tissue homogenates were resuspended in RIPA lysis buffer and centrifuged. Briefly, RIPA Buffer (Thermo Scientific) was added to the tissue homogenates. The mixture was re-suspended thoroughly. The mixture was shaken gently for 15 minutes on ice. It was centrifuged at ~14,000 × g for 15 minutes to pellet the cell debris. The supernatant was transferred to a new tube for further analysis. Total protein concentration was measured by BCA. All samples were diluted to a total protein concentration of 10 mg/ml and CCL22 concentrations were measured by ELISA (Wuhan Biotech). The final chemokine concentration was calculated as nanograms cytokine per miligram of total protein in the respective lysate.

### 2.19 Statistical analysis

Statistical analysis of data was performed either by two-tailed student t-test or two-way analysis of variance (ANOVA) with GraphPad software 6.0. P value of ¾ 0.05 was considered as significant. Data are represented as the mean ± SEM.

## 3 Results

### 3.1 MIP immunotherapy reduces the percentage of immunosuppressive Tregs in the tumor microenvironment

**Figure 1:**
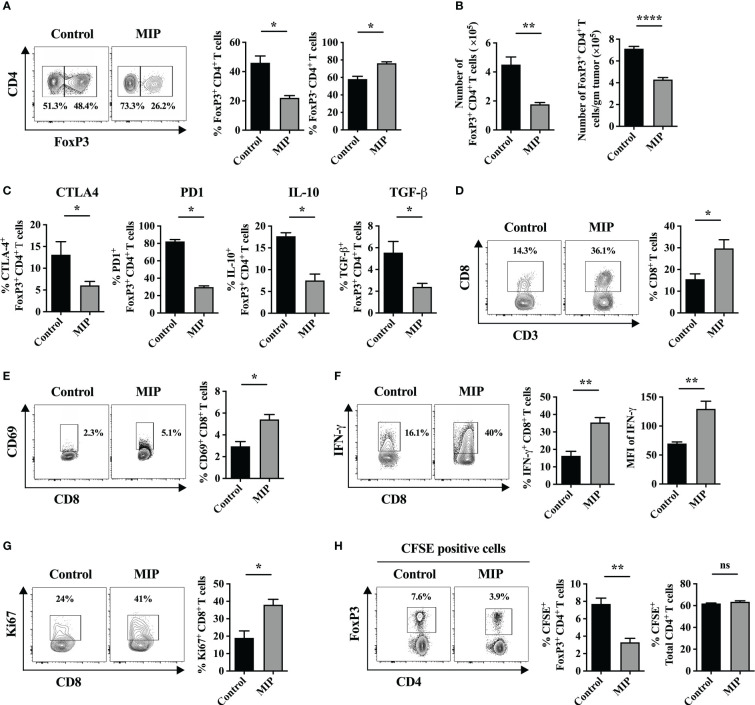
Effect of MIP immunotherapy on the frequency and function of tumor infiltrating Tregs and CD8^+^ T cells: **(A)** Twenty one days post tumor implantation, immune cells were separated from tumor tissue and stained for FoxP3 on live CD3^+^CD4^+^ T cells and analyzed by flow cytometry. The control tumor bearing mice were treated with PBS (vehicle control). The contour plots represent one tumor bearing mouse per group showing the percentage of FoxP3^+^ cells within tumor-infiltrating CD4^+^ T cells. **(B)** (Left) Specific number of tumor-infiltrating CD4^+^ FoxP3^+^ Tregs; (Right) Number of tumor-infiltrating CD4^+^ FoxP3^+^ Tregs per gram of tumor (n=5 mice/group). **(C)** Percent frequency of Tregs expressing CTLA4, IL10, TGF-β and PD1 in Control and MIP treated TME (n=4 mice/group). **(D)** Flow-cytometric analysis of percentage of intratumoral CD8^+^ T cells in control/MIP treated mice. The contour plots represent one tumor bearing mouse per group. **(E)** Percent frequency of activated CD8^+^ T cells in the TME. **(F)** Flow cytometric analysis of IFN-γ producing CD8^+^ T cells in the TME of control and MIP treated mice. **(G)** Percent frequency of potentially proliferating CD8^+^ T cells was determined in the TME by analyzing expression of Ki67 in them. **(H)** To determine whether MIP immunotherapy suppresses Treg migration into the TME, 24 hours after the third dose of MIP, CFSE-labeled splenocytes of healthy donor mice were adoptively transferred by retro-orbital route into the tumor bearing mice. Tumors were resected 18 hours after adoptive transfer, followed by flow cytometric analysis of intratumoral CFSE^+^ Treg cells and CFSE^+^ total CD4^+^ T cells. Representative contour plots showing the frequency of CFSE^+^ Tregs in one mouse per group. All findings are shown as bar graphs that represent the mean ± SEM of five mice per group in one experiment. Statistical significance was determined by unpaired non-parametric student’s t-test (*:p<0.05; **:p<0.01; ****:p<0.0001; ns, non-significant). Two/three independent repeats were done for all experiments.

Immunosuppressive Tregs are essential for inducing immunological tolerance. They have been reported to accumulate in a rising variety of tumors and actively inhibit anti-tumor responses. In order to examine the effect of MIP immunotherapy on tumor infiltrating Tregs, we determined the frequency of these immunosuppressive cells in the TME from the control and MIP treated mice using flow cytometry. We observed that the intratumoral Treg percentage was considerably reduced to almost half in the MIP treated tumors relative to the untreated group (**Figure 1A**). A similar decrease in FoxP3^+^ cell count was also observed when the number of Foxp3^+^ cells was calculated per gram of tumor tissue (**Figure 1B**). Since, over-expression of immune inhibitory checkpoints like CTLA4, KLRG1 and PD1 on Tregs and secretion of immunosuppressive cytokines like IL-10 and TGF-β by them facilitate their suppressive activity (20–22), we further analyzed the phenotype of these intratumoral Tregs and examined whether MIP has any role in regulating the same. It was found that CTLA4, PD1, KLRG1, IL-10 and TGF-β were expressed by a considerable proportion of the total Tregs in the B16F10 TME thereby indicating their highly immunosuppressive nature. Although the percentage of IL-10^+^, TGF-β^+^, CTLA-4^+^ and PD1^+^ subsets of Tregs in the MIP treated group was significantly reduced when compared to the control group (**Figure 1C**), no change was observed in the percent frequency of KLRG1^+^ subset of Tregs (**Figure S1A**). Besides, there was no difference in the mRNA expression levels of these molecules between the two groups when examined on sorted Tregs (data not shown). This suggests that the effect of MIP immunotherapy is restricted to reducing the percent frequency of intratumoral activated Tregs.

The fundamental function of Tregs in TME is to suppress the proliferation of effector anti-tumor T cells (23). So, an *ex-vivo* Treg suppression assay was carried out to assess the effect of MIP immunotherapy on the suppressive function of tumor infiltrating Tregs (Ti-Tregs). Naïve T cells (CD4^+^CD25^−^) were purified from the spleen of tumor bearing mice and labeled with CFSE. These were regarded as responder cells. Regulatory T cells (CD4^+^CD25^+^) were purified from control and MIP treated tumor draining lymph nodes by magnetic-activated cell sorting. CD4^+^CD25^−^ T cells and CD4^+^CD25^+^ Tregs were co-cultured with splenocytes of RAG^-/-^ mice (regarded as antigen presenting cells) in anti-CD3/anti-CD28 coated plate which provided polyclonal stimulation for the naive T cells to proliferate. The suppressive effect of Tregs on the proliferation of naïve T cells was determined. The CFSE dilution experiment revealed that Tregs from both control and MIP treated tumor-bearing mice suppressed naive T cell proliferation at similar levels (**Figure S1B**). Furthermore, there was no difference observed in the mRNA levels of *Il-10* and *Tgf-β* between both the groups (data not shown) when analyzed from the co-cultured cells of Treg suppression assay (equal number of Tregs was present in both the groups). These findings imply that neither the expression of *Il-10* or *Tgf-β* on Ti-Tregs nor the suppressive activity of Tregs is impacted by MIP immunotherapy. It was concluded that reduction in the percentage of total Ti-Tregs in MIP treated TME got reflected in the lower percent frequency of IL-10^+^, TGF-β^+^, CTLA-4^+^ and PD1^+^ Ti-Tregs in the TME.

### 3.2 MIP therapy increases the percent frequency and effector function of CD8^+^ T cells in the

#### 3.2.1 TME

A previously published report demonstrated that a functional immune crosstalk between CD4^+^ and CD8^+^ T cells is crucial in mediating the anti-tumor responses by MIP. The critical role of CD4^+^ T cells and IFN-γ in MIP immunotherapy has also been reported in the same study (23, 24). On the other hand, our group has reported previously that MIP immunotherapy increases the cytotoxic activity of splenic CD8^+^ T cells when cultured *ex-vivo* (15) but not much detail is known about the influence of MIP on intratumoral CD8^+^ T cells. Interestingly, it was observed that MIP treatment increased the frequency of CD8^+^ T cell population in the TME by 40-50% in comparison to un-treated tumors (**Figure 1D**). Furthermore, the activation potential of CD8^+^ T cells also increased significantly upon MIP treatment as there was higher frequency of activated tumor infiltrating CD8^+^ T cells in the TME. The percentage of CD69^+^CD8^+^ T cells increased by 50% compared to the control tumors (**Figure 1E**). We also observed that administration of MIP remarkably restored the effector function of these CD8^+^ T cells as there was significant induction of IFN-γ expression from CD8^+^ T cells and higher percent frequency of IFN-γ^+^ CD8^+^ T cells (**Figure 1F**).

Next, we investigated the reason behind higher percent frequency of intratumoral CD8^+^ T cells within the MIP treated TME. We hypothesized that it could be due to higher proliferation of these cells. So, we analyzed the percentage of Ki67^+^CD8^+^ T cells in the TME. Strikingly, there was significant increase in the percent frequency of Ki67^+^CD8^+^ T cells in the tumors that were treated with MIP (**Figure 1G**).

### 3.3 MIP inhibits migration of Tregs into the TME

Next, we investigated the probable reason for decreased Treg percentage in the TME. To examine whether the decreased frequency of intratumoral Tregs is an outcome of reduced migration of FoxP3^+^ cells into the tumor/due to lesser proliferation/higher apoptosis of Tregs in the TME, we first analyzed the proliferation of the tumor infiltrating Tregs (Ti-Tregs) using Ki67 as the proliferation marker. No change in Ti-Treg specific Ki67 expression was observed in the MIP treated group compared to the control providing evidence that MIP therapy doesn’t affect the proliferation of Ti-Tregs (**Figure S1C**). Further to examine whether increased death or apoptosis of Tregs is contributing to reduced frequency of these cells in the MIP treated TME, we checked the frequency of apoptotic Tregs in the TME using Annexin-V and live-dead dye staining. We found no difference in their proportion, indicating that apoptosis of Tregs is not the cause of their decreased frequency (data not shown).

To analyze the migration of FoxP3^+^ cells into the tumor, twenty-four hours after the third dose of MIP, CFSE-labeled splenocytes (3 x 10^7^/mice) from healthy mice were transferred intravenously. Tumors were resected 18 hours after transfer and CFSE^+^ Treg cells (CFSE^+^CD3^+^CD4^+^FoxP3^+^) were analyzed by flow cytometry. The representative gating strategy used to analyze intratumoral CFSE^+^ Tregs have been demonstrated in **Figure S2**. Interestingly, in the tumors of MIP-treated animals, the fraction of transferred Tregs (CFSE^+^) within intratumoral total CFSE^+^ T cells were reduced to almost half of the percentage of CFSE^+^ Tregs migrated in tumor of control mice. In contrast, no difference in the proportion of transferred Tregs was observed in the spleen (data not shown). There was no decrease observed in the total number of transferred CD4^+^ T cells (CFSE^+^CD3^+^CD4^+^), indicating a specific inhibition of Treg migration in the tumor of MIP treated group (**Figure 1H**). So, reduction in the percentage of Tregs in the TME of MIP treated mice is attributed to the reduced trafficking of Tregs into the TME and this effect was specific to tumor.

### 3.4 MIP therapy downregulates expression of CCL22 on tumor infiltrating dendritic cells to reduce the migration of Tregs into the TME

We next investigated the mechanism involved in MIP mediated reduction in the migration of Tregs in the TME. It has been reported that CD62L expression level is crucial in promoting the trafficking of Tregs from thymus to lymphoid tissues and from lymphoid tissues to non-lymphoid tissues including tumor (25). We found that the percent frequency of CD62L^+^ Tregs as well as expression of CD62L on Tregs is significantly lower in the B16F10 TME (irrespective of MIP treatment) as compared to the splenic Tregs which is consistent with literature (11) (**Figure S3A**). Tregs having low expression of CD62L have been reported to express a wide variety of chemokine receptors on their surface which promote trafficking from lymphoid tissue to various non-lymphoid tissues by interacting with respective chemokines (25, 26). So, we hypothesized that MIP might have a role in regulating the chemokine mediated migration of Tregs into the TME. Hence, we analyzed the expression levels of several major Treg recruiting chemokines and their cognate receptors on tumor infiltrating leukocytes in MIP treated/control mice.

**Figure 2:**
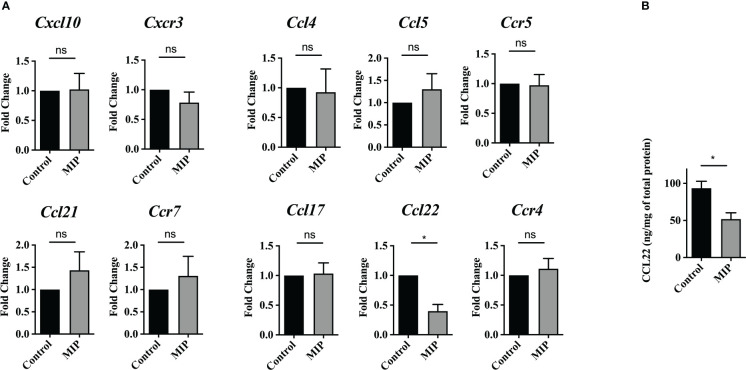
Expression level of intratumoral Treg recruiting chemokines and their cognate receptors: **(A)** mRNA levels of Treg recruiting chemokines and their cognate receptors were analyzed on total tumor infiltrating leukocytes by qRT-PCR (n=4). **(B)** Intratumoral CCL22 levels was determined by ELISA (n=5). All the bar-graphs show mean ± SEM. Statistical significance was determined by unpaired student’s t-test (*:p<0.05, ns, non-significant). Two independent repeats were done for all experiments.

Several chemokines such as CCL5, CCL17, CCL22, CCL21, and CXCL12 have been known to mediate homing of Tregs in the malignant tissue (27). So, we first analyzed mRNA levels of the cognate receptors of major Treg recruiting chemokines on total TILs/sorted Tregs in the MIP treated vs control group. Although there was considerable level of basal expression of *Ccr5, Ccr4, Cxcr3* or *Ccr7* on TILs/sorted Tregs, no difference was found in their expression between the two groups. Next, we analyzed the mRNA expression of the major chemokines on tumor infiltrating leukocytes as well as on the primary tumor cells fraction obtained after density gradient centrifugation. Interestingly, in the MIP treated group tumor infiltrating immune cells had about 60% reduction in the mRNA level of *Ccl22* in comparison to control group (**Figure 2A**). Additionally, the MIP-treated group showed a significant suppression of the intratumoral protein level of CCL22 (**Figure 2B**). None of the other chemokines showed any altered expression in MIP treated group. CCL22 has been reported to be crucial for migration of Tregs in several murine as well as human cancers (28). Several groups have shown that it is one of the major chemokine responsible for increased Treg recruitment in solid tumors and number of infiltrating FoxP3^+^ cells have linear correlation with intratumoral CCL22 levels (29). There are several reports which show that blocking the CCL22/CCL17–CCR4 axis by using specific antibodies, antagonists or siRNA, resulted in remarkable reduction in Tregs and increased anti-tumor responses (30–34). So, the inference from our data is that MIP significantly suppresses the expression of CCL22 on tumor infiltrating immune cells which could be responsible for the impaired (reduced) trafficking of Tregs from the circulation into TME.

Alternatively, in the primary tumor cells obtained from MIP treated group, no difference was observed in the expression of any of these chemokines *Ccl5, Ccl4, Ccl21* and *Cc122* except *Ccl17* which was downregulated approximately by 60-70% (**Figure S3B**). Since the basal expression level of *Ccl17* was very low, it was not considered for further study.

Since, CCL22 was the only chemokine which showed significant change in the expression level after MIP treatment, we next searched for the cellular source/s of CCL22 in the tumor infiltrating leukocytes (TILs). Since, DCs are reported to be the exclusive source of homeostatic CCL22 in the lymph node (35), we isolated CD11c^+^ DCs from tumor draining lymph node using a pan DC isolation kit and did quantitative RT-PCR analysis of expression of *Ccl22* on these DCs whose purity was ~80%. High level of *Ccl22* was observed in the CD11c^+^ DC fraction (**Figure S4A**). A very low level of *Ccl22* was also found in the CD11c^-^ fraction. This is probably expressed by the macrophages which are also known to secrete CCL22 (36). So, it was concluded that tumor-infiltrating CD11c^+^ dendritic cells are the predominant source of CCL22 in the TME of B16F10 melanoma. Further, it was observed that there was significant suppression of DC specific *Ccl22* expression but no change in the *Ccl22* expression on CD11c^-^ fraction in the MIP treated group (**Figure S4B**).

### 3.5 MIP therapy remodulates the immunosuppressive tumor-infiltrating dendritic cells towards immunostimulatory phenotype

**Figure 3:**
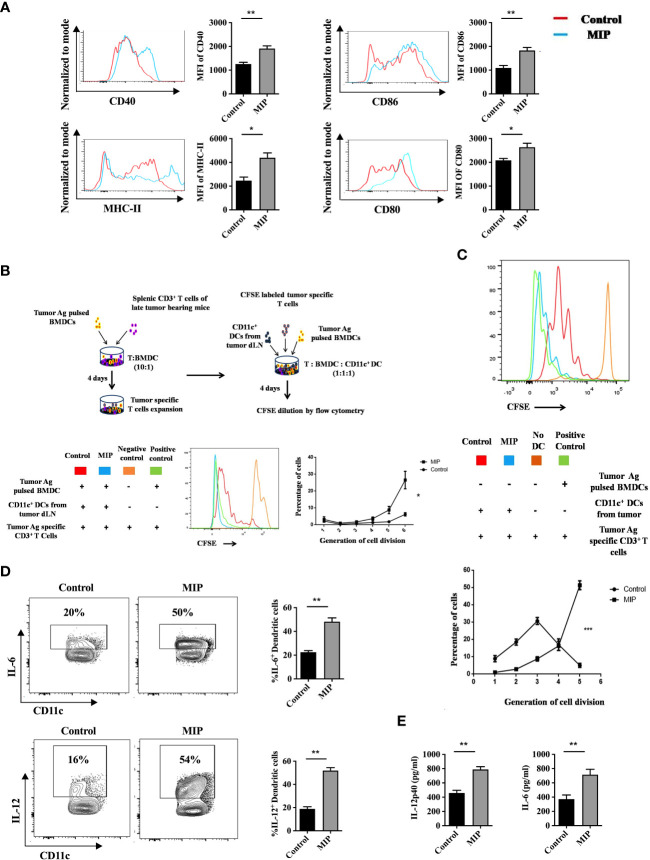
Phenotypic and functional analysis of tumor infiltrating CD11c^+^ dendritic cells: **(A)** Flow cytometric analysis of activation and maturation markers on Ti-DCs including CD80, CD86, MHC-II and CD40. Representative histogram plots indicate MFI of each activation marker in one mouse per group (n=5). **(B)** *Ex-vivo* DC suppression assay: (Top) Tumor-specific CD3^+^ T cells were obtained as depicted in the flow chart. After CFSE labeling, they were added to culture wells containing tumor pulsed BMDCs (1:1). Sorted CD11c^+^ DCs (TI-DC) either from Control or MIP treated tumor was added to these cultures making a 1:1:1 ratio (T cell/pulsed BMDC/sorted DC). (Bottom) The histogram plot is a representative of CFSE dye dilution which indicates tumor specific T cell proliferation of one mouse per group (proliferation induced by tumor pulsed BMDCs in the presence of Ti-DCs). The line graph represents percentage of tumor specific T cells in each generation of cell division (n = 5 mice/group). For the line graph, statistical significance was determined by two-way ANOVA. **(C)** TI-DC mediated T cell proliferation assay: CFSE labeled tumor-specific CD3^+^ T cells were co-cultured with CD11c^+^ DCs sorted either from Control or MIP treated tumor tissue (T cell: TI-DC ~ 10: 1). The line graph represents percentage of tumor specific T cells in each generation of cell division (n = 5 mice/group). For the line graph, statistical significance was determined by two-way ANOVA. (***:p=0.0001 ; *:p<0.05). **(D)** Single-cell suspensions from tumors were stained for CD11c, IL-12 and IL-6; Percent frequency of IL-12 and IL-6 on Ti-DCs were evaluated by flow cytometry. Representative contour plot showing the frequency of IL-12^+^ and IL-6^+^ DCs in one mouse per group. **(E)** In a tumor antigen recall response, IL-12 and IL-6 level was determined (by ELISA) in total leukocytes from tumor draining lymph nodes of control/MIP treated mice. All findings that are shown as bar graphs represent the mean ± SEM of five mice per group in one experiment. Statistical significance was determined by unpaired non-parametric students t-test (*:p<0.05, **:p<0.01, ns, non-significant). For all experiments, two independent repeats were done.

DCs are crucial players in tumor immunotherapy (37). In the TME, tumor antigen presentation by DCs to the effector T cells is an important phenomenon to stimulate anti-tumor response (38). As the tumor progresses, due to the influence of various tumor-derived immunosuppressive factors, DCs lose their anti-tumor activity (39, 40). On stimulation by TLR ligands or other receptor-ligand interactions they get remodulated and actively participate to eliminate the growing tumor cells (41). In previously published reports from our group, we observed that MIP significantly induces the secretion of pro-inflammatory cytokines like IL-12, TNF-α and IL-6 from bone marrow derived DCs. Preliminary results from our lab also indicated that MIP has a potent role in regulating the DC phenotype (15, 16). So, we further evaluated the immunomodulatory effect of MIP on conventional DCs. Higher expression of CD86, CD80, CD40 and MHC-II was observed on DCs of MIP treated group suggesting that MIP treatment remodulated tolerogenic phenotype of the Ti-DCs to activated and matured state (**Figure 3A**). This was further confirmed in an *ex-vivo* DC suppression assay, where influence of MIP treated CD11c^+^ DCs (isolated from tumor draining lymph node) on the capacity of other immunocompetent DCs (tumor antigen pulsed BMDCs) to present tumor antigens to tumor specific T cells was determined. Remarkably, DCs sorted from MIP treated group did not suppress tumor-antigen specific T cell proliferation induced by the immunocompetent BMDCs as there was 60% - 80% increase in the frequency of tumor antigen specific T cells in the later generations of cell division (generation 5 and 6) compared to the control group. This study confirms that DCs from the control TME strongly suppress the tumor specific T cell proliferation which is reversed by MIP immunotherapy (**Figure 3B**).

Next, we hypothesized that appropriate antigen presentation by DCs might be responsible for restoring the effector function and proliferation of CD8^+^ T cells in the TME. So, we analyzed the tumor antigen presenting potential of CD11c^+^ Ti-DCs sorted from the tumor microenvironment of control and MIP treated mice. It was observed that MIP treated Ti-DCs induced notable increase in proliferation of tumor-reactive T cells thereby leading us to conclude that MIP treated Ti-DCs efficiently present tumor antigens to T cells compared to the control Ti-DCs (**Figure 3C**).

In addition, the activation and remodulation of these Ti-DCs by MIP leads to significant upregulation in the expression of IL-12 and IL-6 on these cells, both of which are key players for inducing acute inflammation to confer protective anti-tumor immunity (**Figure 3D**). To investigate whether induction of these cytokines in the MIP treated group is specific to tumor antigens, tumor antigen specific recall response was evaluated. For this, immune cells from tumor draining lymph nodes were stimulated *in vitro* with B16F10 tumor lysate and supernatants were collected for cytokine analysis. Immune cells from MIP-treated mice produced higher amounts of IL-12 and IL-6 upon incubation with tumor lysate as compared to control mice (**Figure 3E**). No recall response was observed when these immune cells were co-incubated with LLC (Lewis lung cancer) lysate thereby proving that higher induction of immune response in MIP treated group is tumor specific (data not shown). In conclusion, the stimulation of Ti-DCs by MIP leads to the induction of the pro-inflammatory circuit in the tumor infiltrating DCs, resulting in eliciting protective anti-tumor responses.

### 3.6 MIP therapy remodulates tumor-resident pDCs towards activated phenotype

**Figure 4:**
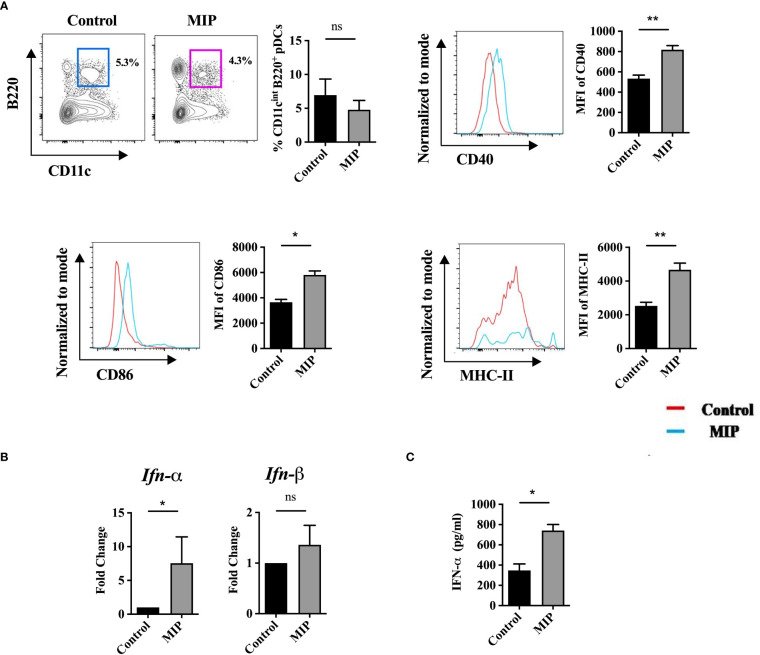
Infiltration of pDCs in B16 tumors and effect of MIP treatment on their phenotype and function: **(A)** Flow cytometric analysis of infiltration, activation and maturation of tumor infiltrating pDCs (n=5). The contour plot is representative image of frequency of pDCs in one mouse per group. Representative histogram plots show MFI of each activation marker in one mouse per group. **(B)** mRNA expression of type 1 IFNs on total tumor infiltrating leukocytes (n=4). **(C)** 10^6^ total leukocytes from tumor draining lymph node were seeded and cultured in complete RPMI for 48 hours in presence of B16 tumor lysate. Level of IFN-α from the supernatants was determined by ELISA (n=5). The results are expressed as mean ± SEM of one experiment. Statistical analysis was performed using unpaired Student’s t test (*:p<0.05, **:p<0.01, ns, non-significant). Two independent experimental repeats were done.

We next investigated the mechanism by which MIP regulates the DCs and T cells to mediate the anti-tumor responses. Previous reports have suggested that the unmethylated cpg containing DNA of MIP is a potent TLR9 ligand (16). Apart from this, MIP is also known to be a TLR2 ligand. MyD88 is the common adapter for all TLRs except TLR3. An observed loss of response to MIP in MyD88^−/−^ DCs indicated that the TLR-MyD88 axis plays a critical role in MIP-mediated DC activation (17). Anti-tumor effect of MIP through the TLR2-MyD88 axis has been well elucidated whereas the TLR9- MyD88 axis has not been studied yet. Although TLR9 is widely expressed in all subsets of DCs but it is predominantly expressed by plasmacytoid DC (pDC) subset (42), so we further studied the effect of MIP on tumor infiltrating pDCs (Ti-pDC). pDCs were selected from the tumor infiltrating leukocytes based on intermediate expressions of CD11c (CD11c^int^), Siglec-H expression (Siglec-H) and co-expression of B220. Since, Siglec-H is a confirmatory marker for pDCs, we first confirmed the presence of Siglec-H^+^ cells in CD11C^int^B220^+^ population of DCs. It was observed that ~80% of tumor infiltrating CD11c^int^B220^+^ DCs expressed Siglec-H thereby confirming the presence of pDCs in the TME of B16F10. Next, we examined whether MIP has any potential to regulate the pDCs. Interestingly, we found higher surface expression of CD40, CD86 and MHC-II on CD11c^int^B220^+^Siglec-H^+^ pDCs in the MIP treated tumors compared to the control tumors. But, surprisingly there was no difference in the frequency of CD11c^int^B220^+^ pDCs in the MIP treated vs control groups (**Figure 4A**). These results provide evidence that MIP has a role in remodulation of tumor resident immunosuppressive pDCs towards functionally active form. Gating strategy used to analyze total Ti-DCs and pDCs in the TME has been included in **Figures S5A, B**, respectively.

Generally, the amount and type of APC-derived cytokines is crucial to shape the immune response in malignancy. Activation of TLR9 in pDCs induces production of predominantly type I IFNs (43). Although we have shown earlier that MIP is a TLR-9 ligand (16, 17) but the role of type 1 IFN has not yet been elucidated in MIP immunotherapy. So, we checked the mRNA levels of the two major types of type 1 IFNs, IFN-α and IFN-β on total TILs. Interestingly, IFN-α expression but not IFN-β was significantly upregulated in the MIP treated group compared to control (**Figure 4B**). Additionally, in a tumor antigen specific recall response, it was further confirmed that the induced secretion of IFN-α in the MIP treated group was specific to tumor antigens (**Figure 4C**). Next, it was also confirmed that the main cellular source of IFN-α in the TME was Ti-pDCs as these cells had the highest expression of IFN-α compared to other immune cell subsets (**Figures S6A, B**). However, there is more room to emphasize in the future on identifying the precise mechanisms through which MIP-treated pDCs govern anti-tumor responses.

### 3.7 Type-I IFN is involved in MIP induced anti-tumor responses *in-vivo*

**Figure 5:**
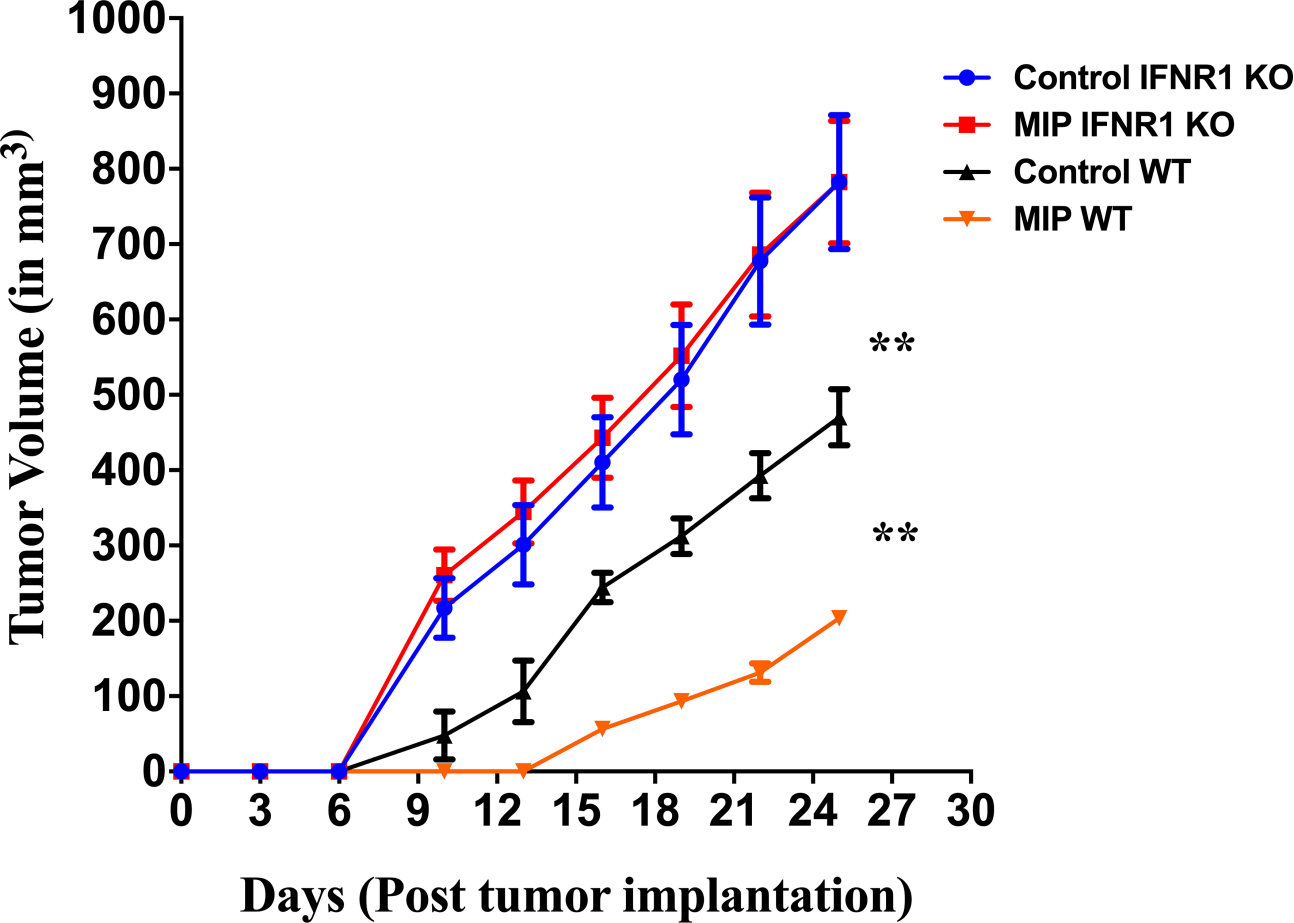
Tumor growth kinetics in wild type and IFNR1^-/-^ mice: 50,000 B16F10 cells were injected in the right flank of wild type (BL/6) and IFNR1^-/-^ mice. MIP was administered peritumorally on day 3, 10, 17 post tumor implantation. Tumor growth was measured every 3 days. Control tumor bearing mice were injected PBS instead of MIP. Tumor volume was calculated using the formula (0.5 x large diameter x small diameter^2^). Data are mean ± SEM values obtained from 8 mice/group. Statistical analysis was performed using two-way ANOVA. (**:p<0.01) Three independent experiments have been repeated.

Type I IFNs are crucial players in regulating adaptive T cell responses as these IFNs induce DC maturation and activation, which in turn promote activation and enhanced effector T cell responses (43, 44). To further confirm the role of type-I IFN in anti-tumor response of MIP immunotherapy in B16F10 melanoma model, we used IFNR1^-/-^ mice in which type-1 IFN receptor has been removed genetically and hence the type-1 IFN signaling is impaired. Wild-type and IFNR1^-/-^ mice were implanted with B16F10 tumor cells, treated with MIP and monitored for tumor growth. In the MIP treated wild type mice, there was significant delay in the appearance of tumor and hence the tumor volume was lower compared to PBS-treated control animals. However in IFNR1^−/−^ mice, the antitumor effect of MIP therapy was dramatically diminished. Tumor volume in MIP-treated IFNR1^-/-^ mice was similar to the tumor volume of control IFNR1^-/-^ animals (**Figure 5**). Additionally, it was also observed that the tumor volume in IFNR1^-/-^ mice was significantly higher than that observed in the wild-type mice with or without MIP treatment. Taken together, these results demonstrated the critical role of type-I IFN signaling in the antitumor response of MIP therapy.

### 3.8 MIP mediated activation of Ti-DCs is dependent on type 1 IFN

**Figure 6:**
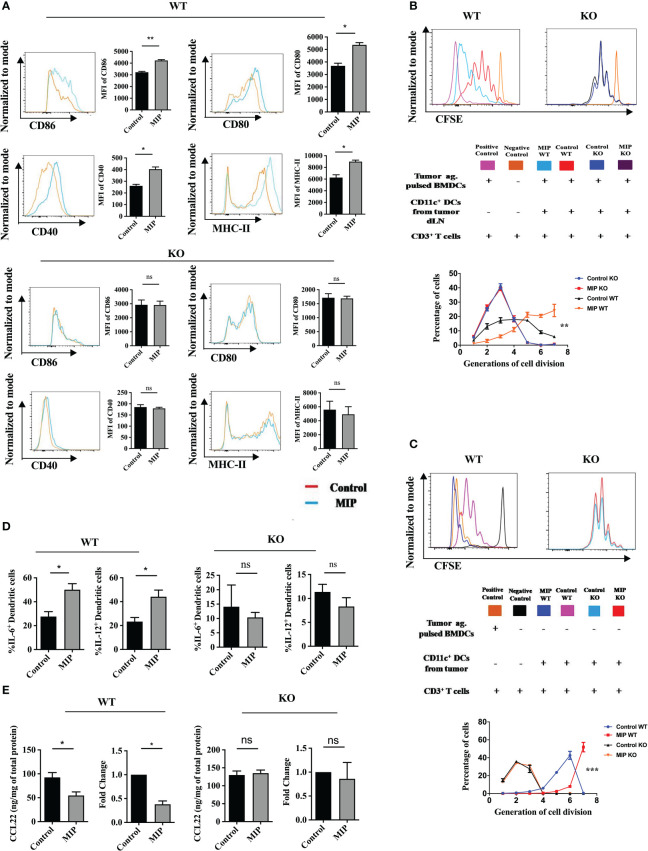
Role of type 1 IFN in remodulation of Ti-DCs in the MIP treated TME: **(A)** Activation and maturation markers on Ti-DCs including CD80, CD86, MHC-II and CD40 were analyzed by flow cytometry in wild type and IFNR1^-/-^ mice. Representative histogram plots indicate MFI of each activation marker (n = 5). **(B)** *Ex-vivo* DC suppression assay: CFSE labeled tumor-specific CD3^+^ T cells were obtained as described earlier. These were added to culture wells containing tumor pulsed BMDCs (1:1). Sorted Ti-DCs either from Control or MIP treated tumor of wild type or IFNR1^-/-^ mice were co-incubated with these cultures in a 1:1:1 ratio (T cell/pulsed BMDC/sorted DC). The histogram plot is a representative of CFSE dye dilution which indicates tumor specific T cell proliferation of one mouse per group (proliferation induced by tumor pulsed BMDCs in the presence of WT or IFNR1^-/-^ Ti-DCs). The line graph represents percentage of tumor specific T cells in each generation of cell division (n = 5 mice/group). For the line graph, statistical significance was determined by two-way ANOVA. (***:p=0.0001 ; **:p<0.01) **(C)** Ti-DC mediated T cell proliferation assay: CFSE labeled tumor-specific CD3^+^ T cells were co-cultured with Ti-DCs sorted from tumor bearing wild type or IFNR1^-/-^ mice treated with or without MIP (T cell: TI-DC ~ 10: 1) (n = 4 mice/group). **(D)** The importance of type 1 IFN in inducing the function of Ti-DCs after MIP immunotherapy is demonstrated by the production of IL-12 and IL-6 from Ti-DCs. **(E) A**fter MIP treatment, mRNA levels of *Ccl22*was determined from the tumor infiltrating DCs of WT and IFNR1^-/-^ mice (n = 4); Intratumoral levels of CCL22 was also analyzed by ELISA in all the four groups (n = 5). All the bar graphs represent results as means ± SEM of one experiment. Statistical analysis was performed using unpaired Student’s t test (*:p<0.05, **:p<0.01, ns, non-significant). Two independent experimental repeats were generated.

Dendritic cells are generally the prime targets of endogenous type-I IFN which leads to tumor growth inhibition (45). So, we hypothesized that the immunomodulatory effect of MIP on conventional DCs might be dependent on type-1 IFN. Interestingly, it was observed that in the MIP treated wild type Ti-DCs, activation and maturation markers including CD80, CD86, CD40 and MHC-II had higher MFI as compared to the control group. Thus, we concluded that MIP therapy significantly increased the activation and maturation of Ti-DCs in the WT mice. Interestingly, MIP immunotherapy lost the potential to activate Ti-DCs in the absence of type 1 IFN signaling (**Figure 6A**). Furthermore, in the *ex-vivo* DC suppression assay (described in **Figure 3B**), DCs sorted from MIP treated wild type tumor draining lymph nodes did not exert significant suppression on the tumor-specific T cell proliferation induced by the immunocompetent BMDCs whereas the control wild type DCs strongly suppressed the tumor specific T cell proliferation. However, Ti-DCs isolated from MIP treated IFNR1^-/-^ tumor draining lymph nodes lacked the potential to actively promote the proliferation of T cells induced by the BMDCs. Instead, these immunosuppressed IFNR1^-/-^ Ti-DCs from both control and MIP treated groups showed inherently diminished immune-stimulatory effect compared to the wild type Ti-DCs (**Figure 6B**). Additionally, the tumor antigen presenting potential of IFNR1^-/-^ CD11c^+^ Ti-DCs to T cells was significantly impaired even after MIP treatment (**Figure 6C**). Therefore, it is concluded that type 1 IFN signaling has an important role in MIP mediated remodulation of the immunosuppressive phenotype of the Ti-DCs to immune-stimulatory phenotype.

Also, administration of MIP dramatically induced the production of pro-inflammatory cytokines IL-12 and IL-6 in wild type intratumoral dendritic cells compared to control whereas the IFNR1^-/-^ DCs were found to be refractory to this effect of MIP immunotherapy (**Figure 6D**). These observations led us to infer that the type-1 IFN signaling is one of the major pathways involved in the phenotypic and functional activation of Ti-DCs in MIP treated mice. In addition, it has been reported that IFN-α/β downregulates the production of CCL17 and CCL22 by splenic DCs (45, 46) which play an important role in Treg migration. So, we investigated whether the MIP mediated downregulation of *Ccl22* on DCs is dependent on type 1 IFN mediated signaling. Strikingly, MIP lost the potential to suppress intratumoral CCL22 in the absence of type 1 IFN signaling. This demonstrated that type 1 IFN signaling is involved in the downregulation of CCL22 expression on tumor infiltrating DCs in MIP immunotherapy (**Figure 6E**)

### 3.9 Type 1 IFN signaling is essential for MIP mediated reduction of Tregs migration into the TME

**Figure 7:**
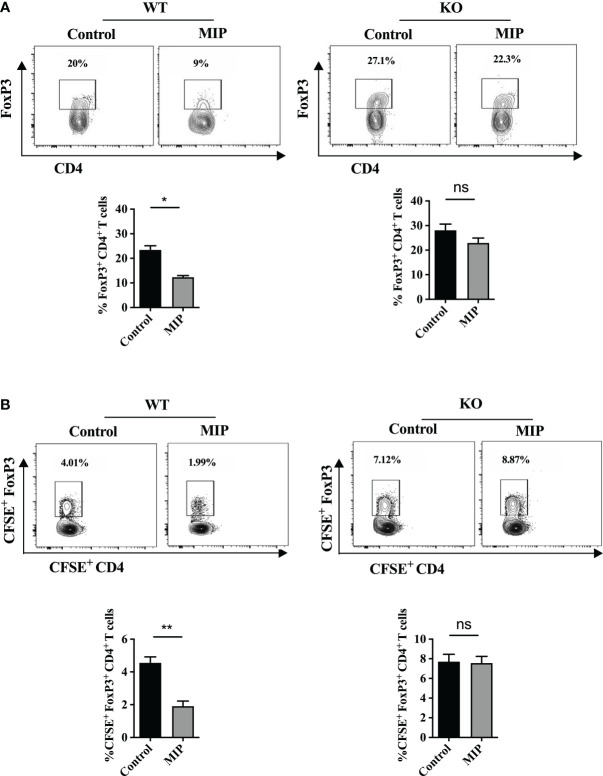
Role of type 1 IFN in regulation of infiltration of Tregs into the MIP treated TME: **(A)** The percentage of Tregs in IFNR1^-/-^/wild type mice in tumors treated with or without MIP. **(B)** Tumor bearing wild type and IFNR1^-/-^ animals, 24 hours after the third dose of MIP, received an intravenous transfer of CFSE-labeled splenocytes of healthy wild type donor mice. Tumors were resected 18 hours after transfer, followed by flow cytometric analysis of intratumoral CFSE^+^ Treg cells. Proportion of CFSE^+^CD4^+^FoxP3^+^ Tregs within the total live CFSE^+^ cells was analyzed in the tumor of untreated/MIP treated mice. Flow cytometry contour plot is shown for one representative mouse per group. The bar graphs represent average of 5 mice per group. The results are expressed as mean ± SEM. Statistical analysis was performed using unpaired Student’s t test (*:p<0.05, **:p<0.01, ns, non-significant). Each experiment was repeated three times independent of each other.

To study whether reduced migration of Tregs in tumors of MIP treated group is dependent on type 1 IFN mediated signaling, tumors were implanted in WT and IFNR1^-/-^ mice treated with or without MIP. The percent frequency of Ti-Tregs and the trafficking of Tregs into the TME were studied. It was found that administration of MIP dramatically lowered the frequency of Ti-Tregs in the WT TME, while it had no effect on the frequency of Tregs in IFNR1^-/-^ TME (**Figure 7A**). These data suggest that type 1 IFN signaling plays a key role in decreasing Treg frequency within tumors of MIP treated group. Another important observation was that MIP influences the trafficking of Tregs into the TME through type 1 IFN signaling. In the tumors of MIP-treated wild type animals, the fraction of transferred CFSE^+^ Tregs within intratumoral CFSE^+^ CD4^+^ T cells was reduced significantly. In contrast, no difference in the proportion of adoptively transferred Tregs was observed in the tumors of IFNR1^-/-^ mice treated with or without MIP (**Figure 7B**). These results provide evidence that MIP immunotherapy inhibits Treg migration into the TME through type 1 IFN signaling.

### 3.10 Type 1 IFN is important for enhanced CD8^+^ T cell function in MIP treated tumors

**Figure 8:**
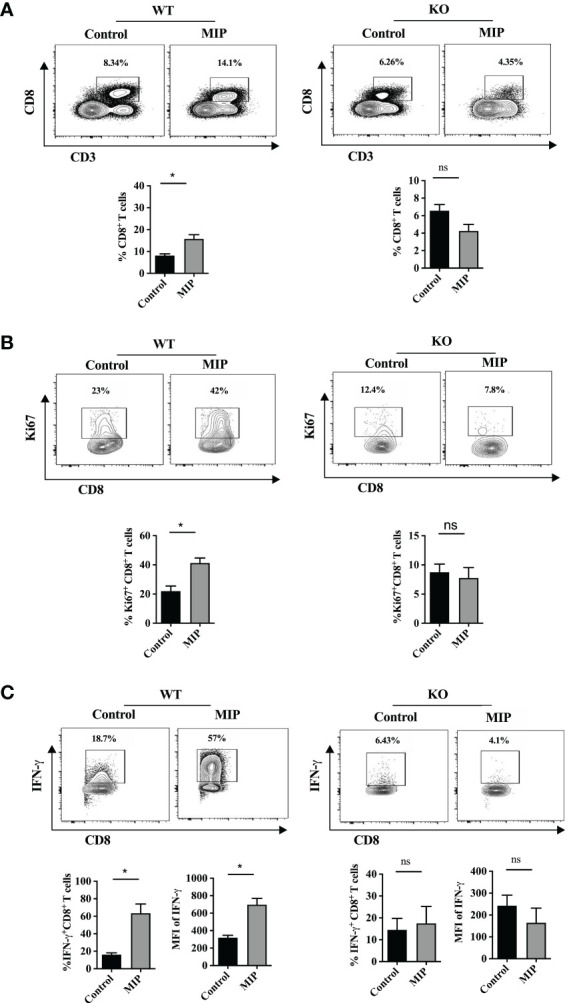
Importance of type 1 IFN in MIP mediated remodulation of percent frequency and effector function of intratumoral CD8^+^ T cells: **(A)** Percent frequency of intratumoral CD8^+^ T cells was determined in wild type and IFNR1^-/-^ tumor bearing mice treated with or without MIP. **(B)** Effect of MIP immunotherapy on proliferation of tumor-infiltrating CD8^+^ T cells in wild type and IFNR1^-/-^ mice. **(C)** In wildtype and IFNR1^-/-^ mice, effect of MIP therapy on effector function of intratumoral CD8 T cells was determined in terms of production of IFN-γ from CD8^+^ T cells. Flow cytometry contour plot is shown for one representative mouse per group. The bar graphs represent average of 5 mice per group. The results are expressed as means ± SEM. Statistical analysis was performed using unpaired non-parametric Student’s t test (*:p<0.05, ns, non-significant). Each experiment was repeated two times independent of each other.

As it was observed that in the absence of type 1 IFN signaling, MIP treatment failed to activate and mature Ti-DCs which were having immunosuppressed phenotype, so we hypothesized that it would also regulate CD8^+^ T cell proliferation and function. So, immune cells were isolated from tumors excised from WT and IFNR1^-/-^ mice and analyzed for proliferation and functional status of CD8^+^ T cells. As expected it was found that in IFNR1^-/-^ mice, frequency of actively proliferating i.e. Ki67^+^CD8^+^ T cells were similar in both MIP treated and control groups (**Figures 8A, B**). This was consistent with our observation in the *ex-vivo* DC suppression assay where the IFNR1^-/-^ DCs even after MIP treatment, significantly suppressed the proliferation of tumor specific T cells induced by the immunocompetent tumor pulsed BMDCs. It was also observed that peritumoral injection of MIP significantly increased the percentage of effector CD8^+^ T cells (IFN-γ^+^CD8^+^ T cells) as well as the production of IFN-γ from CD8^+^ T cells in the wild type mice whereas these effects were diminished in the IFNR1^-/-^ mice (**Figure 8C**). Thus, we concluded that MIP mediated activation of type 1 IFN signaling plays an essential role in restoring the proliferation and effector function of tumor infiltrating CD8^+^ T cells.

## 4 Discussion

In this study, we delineated the underlying mechanism by which MIP modulates the innate and adaptive immune responses to effectively reduce the growth of B16F10 tumor. Previous studies from our group have shown that peritumoral administration of MIP induces Th1 type anti-tumor responses in the TME (15). However, this study has demonstrated for the first time significance of type 1 IFN signaling in eliciting MIP-mediated anti-tumor responses. We found that MIP immunotherapy drastically reduced the infiltration of Tregs into the TME and substantially enhanced the activation and function of intratumoral plasmacytoid DCs (pDCs), conventional DCs and CD8^+^ T cells in a type 1 IFN dependent pathway. In order to successfully establish a tumor, the growing tumor cells and the surrounding cells secrete various cytokines and chemokines to recruit immunosuppressive cells which convert the TME into a suppressive state (47). For effective elimination of the growing tumor, it is important to limit the frequency or the activity of these immunosuppressive cells. Tregs are one of the major subsets of immunosuppressive cells recruited to the tumor microenvironment which downmodulates anti-tumor response within the TME. We have observed that peritumoral administration of MIP significantly reduced the frequency of activated intratumoral Tregs compared to the control group. As there was no change in the percent frequency of KLRG1+ Tregs in the MIP treated group, it suggests that the frequency of these short lived, terminally differentiated Tregs is not influenced by MIP immunotherapy (22, 25, 48). However, further extensive investigation is required to get a specific conclusion.

When the underlying mechanism responsible for lower percentage of Tregs in the TME was investigated, it was found that TiTregs doesn’t have reduced proliferation or higher apoptosis. Nonetheless, MIP treatment significantly downregulated expression of CCL22 on dendritic cells, a chemokine majorly responsible for recruiting Tregs in tumors. Reduced Treg frequency was associated with lower levels of immunosuppressive cytokines and inhibitory checkpoint proteins in the TME. Thus, our findings imply that MIP immunotherapy, by inhibiting CCL22-mediated Treg recruitment into the TME, drastically alters the immunosuppressive environment towards an immunogenic one. This in turn provided the milieu which converted tumor-infiltrating DCs and T cells to functionally active phenotype.

Antitumor immunity is strongly influenced by the ratio of tumor antigen-specific effector CD8^+^ T cells and regulatory T cells (47, 49). In addition to lowering the recruitment and activity of immunosuppressive cells, it is crucial to generate tumor specific CTLs for effective ablation of the proliferating tumor cells. Although CTLs can successfully infiltrate and kill the tumor cells during early tumor growth, they gradually lose their cytotoxic ability under constant influence of the immunosuppressive factors derived from the growing tumors. Therefore, they exhibit a functionally compromised effector phenotype. But, induction of a proper immune crosstalk between innate cells and T cells could successfully reverse the tumor associated CD8^+^ T cell dysfunction. In the MIP treated group, it was observed that a significantly higher percentage of activated and proliferating CTLs were present. The effector function of these cells was also restored remarkably in the MIP treated group. Thus, our data suggests that MIP mediated anti-tumor responses involve a high density of activated CD8^+^ T cells along with substantial reduction in the percentage of T_reg_ cells in the TME which resulted in crucial shift in the ratio of effector CD8^+^ T cells to T_reg_ cells in the favour of CD8^+^ T cells. In this study, type I IFN signaling was established as a key player in mediating the anti-tumor effects of MIP immunotherapy. IFN-α is a key pleiotropic cytokine that promotes neo-vascularization, tumor cell killing and efficient immune cell function (50). Additionally, it restores the anti-tumor effector function of immune cells that were rendered immunosuppressive by the influence of anti-inflammatory factors released by tumors (51). MIP treatment significantly increased the intratumoral levels of IFN-α in comparison to control. This prompted us to check whether type 1 IFN signaling has any contribution in mediating the anti-tumor responses by MIP. Interestingly, it was observed in the absence of intact type 1 IFN signaling, MIP immunotherapy loses its anti-tumor potential including i) suppression of CCL22 expression on tumor infiltrating DCs, ii) reduced migration of Tregs into the TME and iii) restoration of proliferation/effector function of intratumoral CD8^+^ T cells. Several studies have reported the important role of type 1 IFNs in promoting cross-priming of CD8^+^ T cells and enhancing CD8 effector T cell expansion, survival and memory transition (52–54). Our study suggests that the increased proliferation and effector function of CD8^+^ T cells is attributed to effective antigen presentation by the MIP treated Ti-DCs.

It was observed that type I IFN signaling was required for MIP mediated induction of Ti-DC maturation and antigen presenting function, in accordance with a similar study claiming that DCs are the relevant targets of type 1 IFN during T cell mediated anti-tumor response (55, 56). Consistent with this, our data also suggests that DCs are the key players in aiding type IIFN driven anti-tumor responses, initiated after MIP treatment. In the presence of effective type 1 IFN signaling pathway, MIP immunotherapy remodulates the otherwise immunosuppressed Ti-DCs towards activated and matured form as they are likely rendered refractory to the suppressive effect of the tumor-derived anti-inflammatory molecules. Additionally, re-modulation of their polarity from immunologically cold to hot, greatly enhanced their effector function as well as the capacity of influencing other immunocompetent DCs in the tumor to induce protective anti-tumor responses. Interestingly, this was dependent on type 1 IFN signaling because Ti-DCs isolated from IFNR1^-/-^ mice showed diminished response even in the presence of MIP immunotherapy. Although the proliferation read out for total T cells was shown in the DC suppression assay, we found that both CD4^+^ and CD8^+^ T cells proliferated substantially higher than the negative control in all the four groups of mice, demonstrating that BMDCs presented tumor antigen to both CD4^+^ T cells and CD8^+^ T cells in an unbiased way, *ex-vivo*. Thus, our study provide evidences that induction of type I IFN by MIP in the TME shapes the appropriate microenvironment for inducing pro-inflammatory responses (Th1 responses) in Ti-DCs. Furthermore, we observed that MIP treatment significantly increased the production of pro-inflammatory cytokines IL-12 and IL-6 from tumor infiltrating DCs in a type 1 IFN dependent pathway. This aspect is specifically important as pro-inflammatory cytokines are essential cytokines involved in eliciting appropriate anti-tumor responses. IL-12 has potent T cell activating properties (57). IL-12 production within tumors plays an important role in shaping the tumor milieu. Delivery of rIL-12 into tumors orchestrates significant tumor regression by inhibiting TGF-β signaling in the TME which in turn activates CTLs (58, 59). Another pro-inflammatory cytokine, IL-6 polarize the tumor microenvironment towards Th1 type. IL-6 has beneficial effects on survival, differentiation, recruitment and proliferation of effector leukocytes in the TME (60). IL-6 signaling shifts the balance in the tumor microenvironment from being pro-tumorigenic to anti-tumorigenic. Although our study suggests the role of type I IFN mediated restoration of immunogenic function of host DCs to regulate CD8^+^ T cells function, the direct effect of type 1 IFNs on T cells may also be required for functioning efficiently (61).

Type 1 IFN dependent suppression of CCL22 and Treg migration is supported by several reports published previously (29, 46). In the context of MIP immunotherapy, our findings reveal type-1 IFN dependent CCL22 suppression and consequently reduced Treg migration as a so far unknown mechanism involved in MIP mediated anti-tumor activity. Prior studies have reported that IFN-α negatively regulates CCL22 expression in tumor infiltrating leukocytes. In another independent study, it has been shown that IL-12 significantly suppresses CCL22 expression in tumors (62). Furthermore, both IL-12 and IL-6 have been known to reduce the infiltration of Tregs into the TME (57, 63). Interestingly, the current study demonstrates MIP to be a potent inducer of IL-12 and IFN-α, pointing to the possibility that MIP might be regulating CCL22 through these cytokines. However, whether these cytokines act individually or synergistically in suppressing dendritic cells specific CCL22 expression in the MIP treated B16F10 TME requires further investigation. In addition, there is a possibility of another scenario going on in the TME; the increase in the production of IFN-γ from the activated CD8^+^ T cells may also have a role in inhibiting the induction of Tregs (64, 65). Taken together, these observations suggest that in MIP immunotherapy, type I IFNs play a pivotal role in promoting adaptive immune responses through the innate immune cells like DCs.

We have previously reported that MIP is a potent TLR-2 and TLR-9 ligand (16). It strongly induces activation of the innate immune cells and through the MyD88 pathway regulates the adaptive counterpart (17). In pDCs, TLR-9 stimulation predominantly produces IFN-α in a MyD88 dependent pathway (66, 67). Our study, for the first time, reports the effect of MIP on pDCs. Several studies have shown that the intratumoral pDCs have immunotolerant and less activated phenotype which contributes to tumor progression (68). However, when they are re-modulated by TLR7/TLR9 ligands, their tolerogenic phenotype gets shifted to activated form and mediate effective anti-tumor immunity through the activation of myeloid DCs, NK cells, and CD8^+^ T cells in solid tumors (69, 70). In our study, pDCs showed higher activation and better effector function in the TME of MIP treated mice but no change was observed in their frequency or infiltration in the tumor, suggesting a potent role of MIP in remodulation of the phenotype and function of the tumor resident pDCs. MIP treated pDCs showed higher expression of IFN-α compared to the control untreated mice and they are the major source of IFN-α in the B16F10 TME. This could be attributed to the uptake of MIP by pDCs followed by interaction with endosomally located TLR9 and induction of IFN-α through MyD88 pathway (71).

So, based on our observations we proposed a model depicting the mechanism involved in MIP mediated anti-tumor responses (**Figure S7**). It shows MIP treatment induces IFN-α secretion from pDCs which in turn results in activation and maturation of Ti-DCs. Activated tumor infiltrating DCs secrete higher amount of IL-12 and IL-6. As a consequence, tumor antigen presentation to CD8^+^ T cells is also enhanced. In addition, the same activated Ti-DCs also suppress CCL22 mediated migration of Tregs into the TME. These immunological modulations within the MIP treated tumor microenvironment are responsible for the reduced tumor growth. However, additional confirmatory studies are required to determine if type 1 IFN signaling has a role in MIP-mediated regulation of other immune cells such as CD4^+^ T cells, macrophages and NK cells, considering that both MIP and type 1 IFN signaling modulate these immune cells (15–17, 72, 73).

## Data availability statement

The original contributions presented in the study are included in the article/**Supplementary Material**. Further inquiries can be directed to the corresponding author.

## Ethics statement

The animal study was reviewed and approved by Animal Ethics Committee of the National Institute of Immunology (IAEC#558/20), (IBSC#403/20).

## Author contributions

GR, SanB and SouB contributed to conceptualization of the study. GR and SanB analyzed and interpreted the data. GR, AC & CA have performed the experiments. GR, AC, BS, LP and SanB have contributed to manuscript preparation. All authors contributed to the article and approved the submitted version.
