# Supplementary material for: Type 1 interferon mediated signaling is indispensable for eliciting anti-tumor responses by Mycobacterium indicus pranii
Source: Front Immunol. 2023 Apr 14;14:1104711. doi: 10.3389/fimmu.2023.1104711 (PMC10140407; doi:10.3389/fimmu.2023.1104711)
Supplement: Supplementary file 1 [file DataSheet_1.docx]

**Supplementary Figures**

**
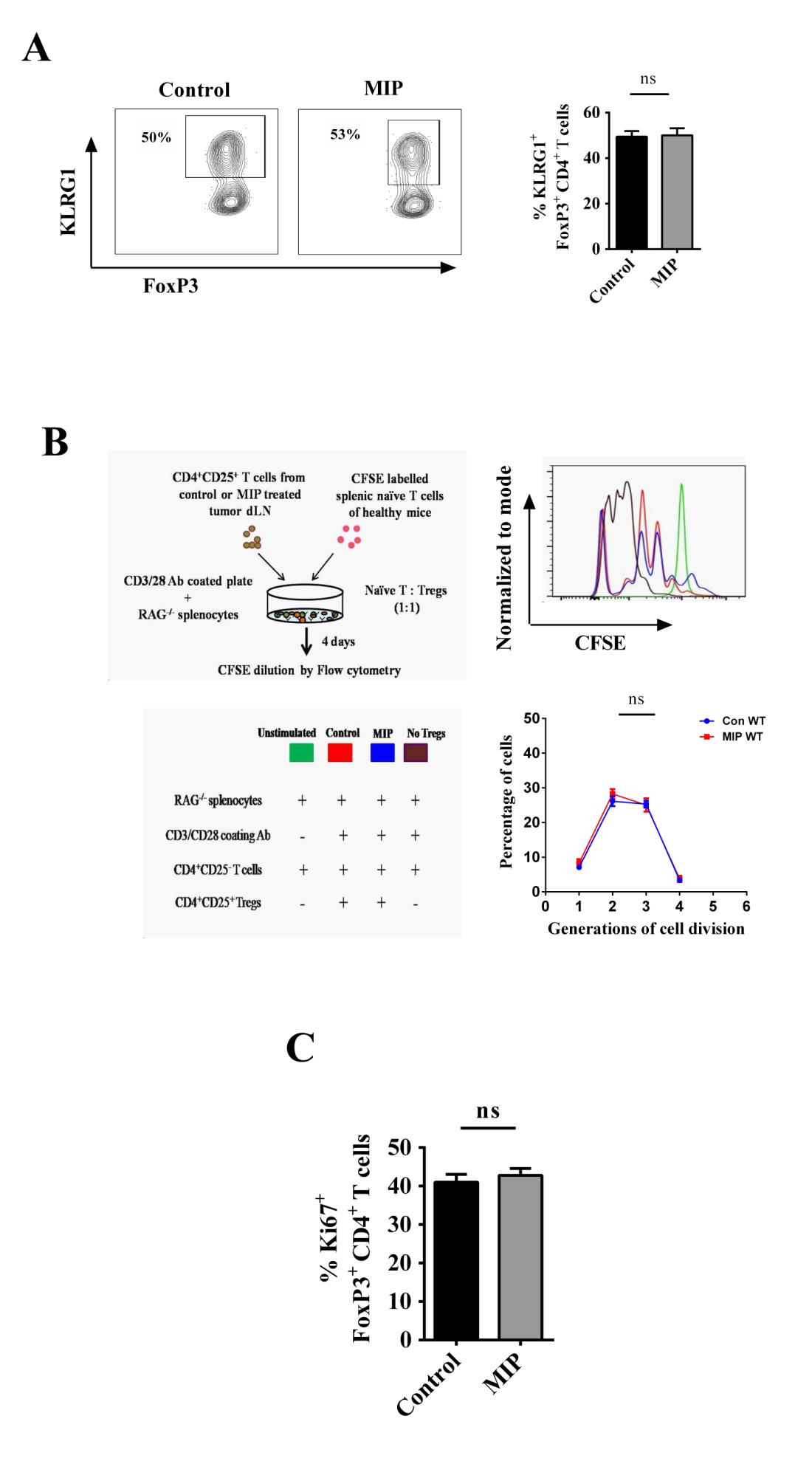
**

**Figure S1. Effect of MIP on proliferation and suppressive function of Tregs. (A)** Percent frequency of Ti-Tregs expressing KLRG1 in Control and MIP treated TME (n=4 mice/group).  **(B) Treg suppression assay**: Splenic naive T cells from tumor bearing mice were labeled with CFSE. These were added to culture plates coated with CD3/CD28 coating antibody (5 μg/ml); to this, splenocytes of healthy RAG^-/-^ mice which will behave as APCs and CD4^+^CD25^+^ Tregs from the tumor draining lymph nodes of control or MIP treated mice were added. 4 days later, naïve T cell proliferation was analyzed in terms of CFSE dilution. Negative control was naïve T cells seeded in uncoated well i.e. no polyclonal stimulation by CD3/CD28 antibody. Positive control was naïve T cells added to antibody coated plate containing only RAG^-/-^ splenocytes and no Tregs; (Right) The histogram plot represents CFSE dye dilution in one mouse per group (n=3) and the line graph represents percentage of T cells in each generation of cell division. For the line graph, statistical significance was determined by two-way ANOVA. **(C)** Proportion of actively proliferating Tregs (Ki67^+^CD4^+^FoxP3^+^) was analyzed in the TME of untreated / MIP-treated mice. The bar graphs represent average of 4/5 mice per group. The results are expressed as means ± SEM. Statistical analysis was performed using unpaired non-parametric Student’s t test.

**
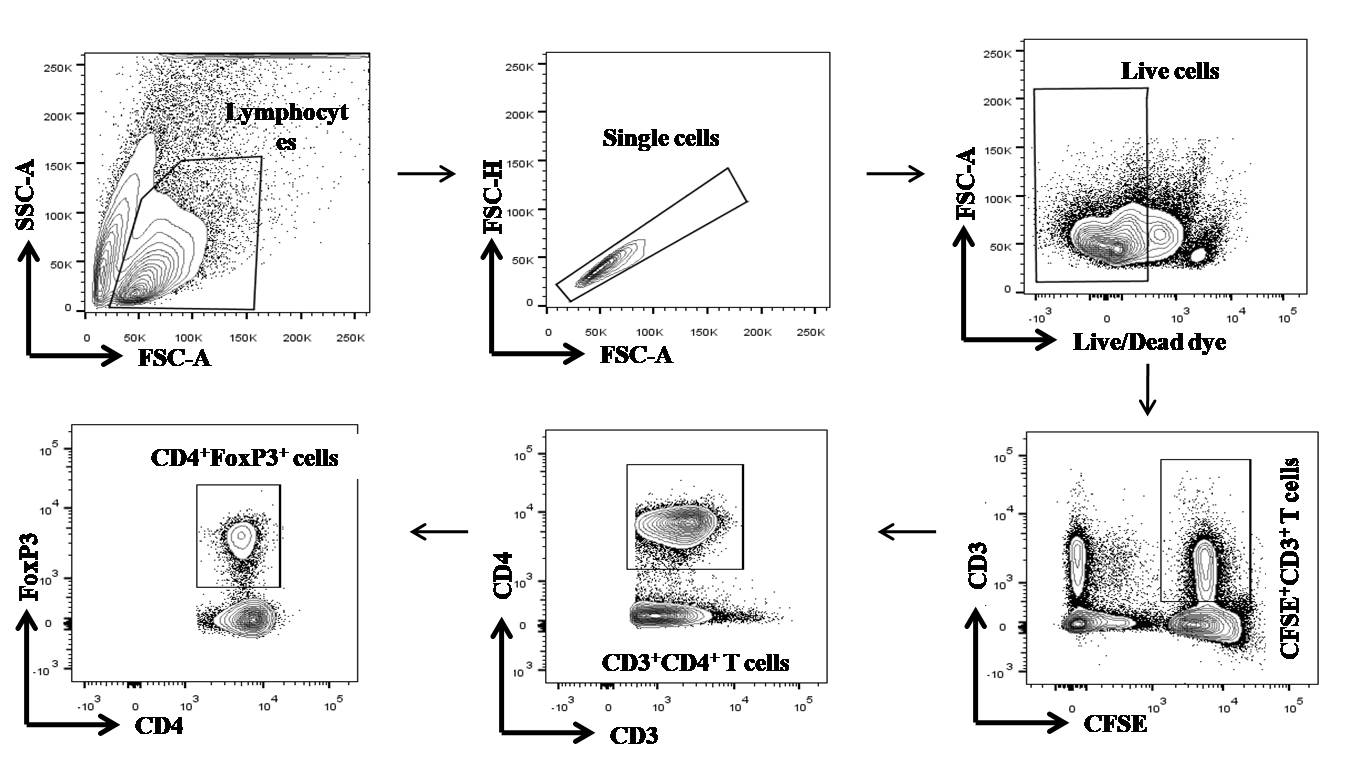
**

**Figure S2. Gating strategy for analyzing adoptively transferred regulatory T cells.** Total splenocytes from naive donor mice were labeled with CFSE and adoptively transferred through retro-orbital route to the tumor bearing mice. After 18h of adoptive transfer, tumors were dissected and single-cell suspension was prepared followed by flow-cytometric analysis in which lymphocytes were selected on the basis of size by plotting FSC-A against SSC-A. Followed by discrimination of single cells based on FSC-A and FSC-H, live cells were selected as Live-Dead dye negative cells. Live cells were further sub-gated for CFSE^+^ tumor infiltrating T cells. Further gated for Tregs (CD4^+^FoxP3^+^ cells).

**
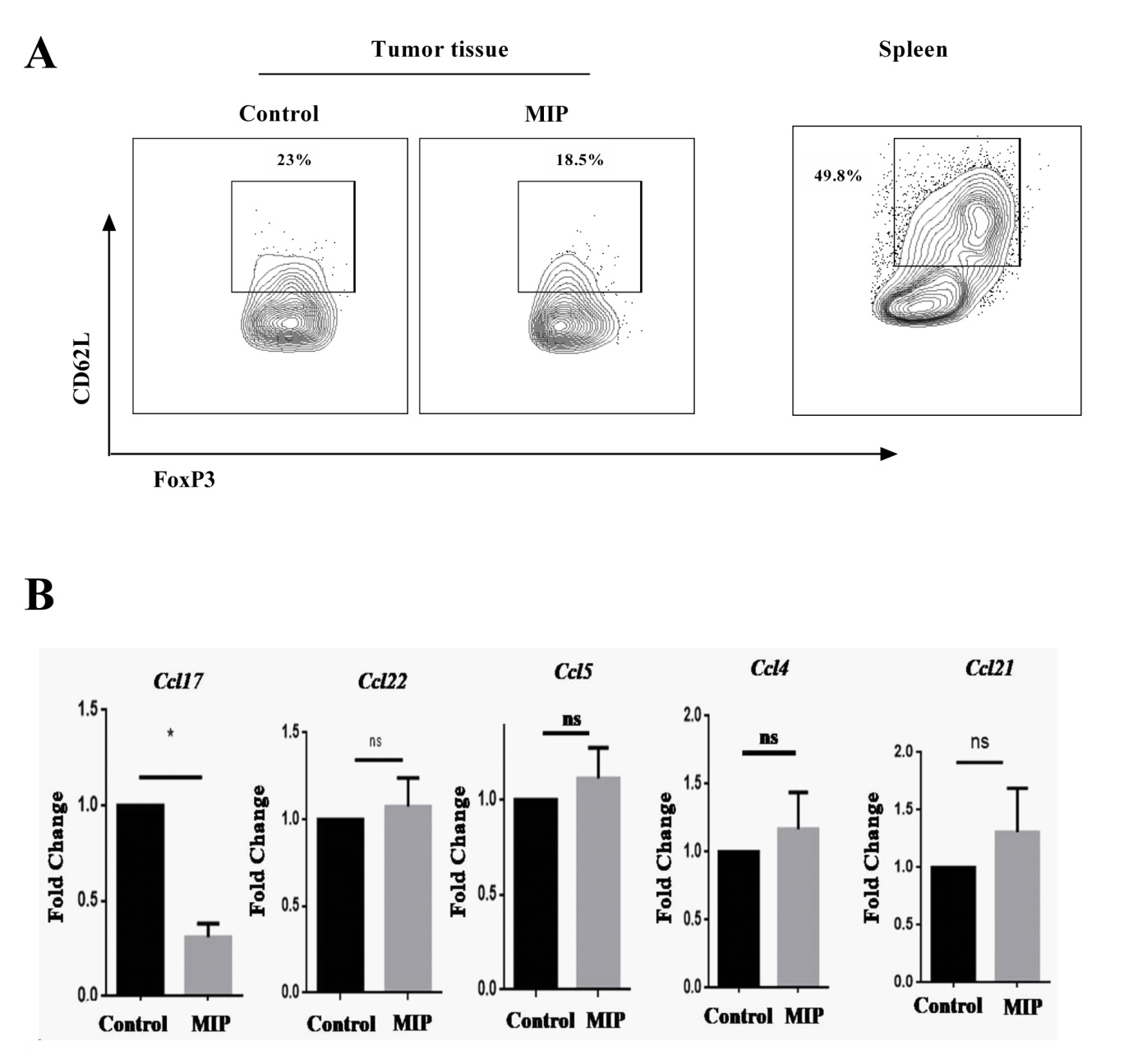
**

**Figure S3. Expression of CD62L on Tregs in tumor/spleen and chemokines on primary tumor cells: (A)** Percent frequency of Tregs expressing CD62L in spleen and Control vs MIP treated TME **(B)** mRNA expression of Treg recruiting chemokines on primary tumor cells (n=4). The bar graphs represent average of all mice per group. The results are expressed as means ± SEM. Statistical analysis was performed using unpaired non-parametric Student’s t test.


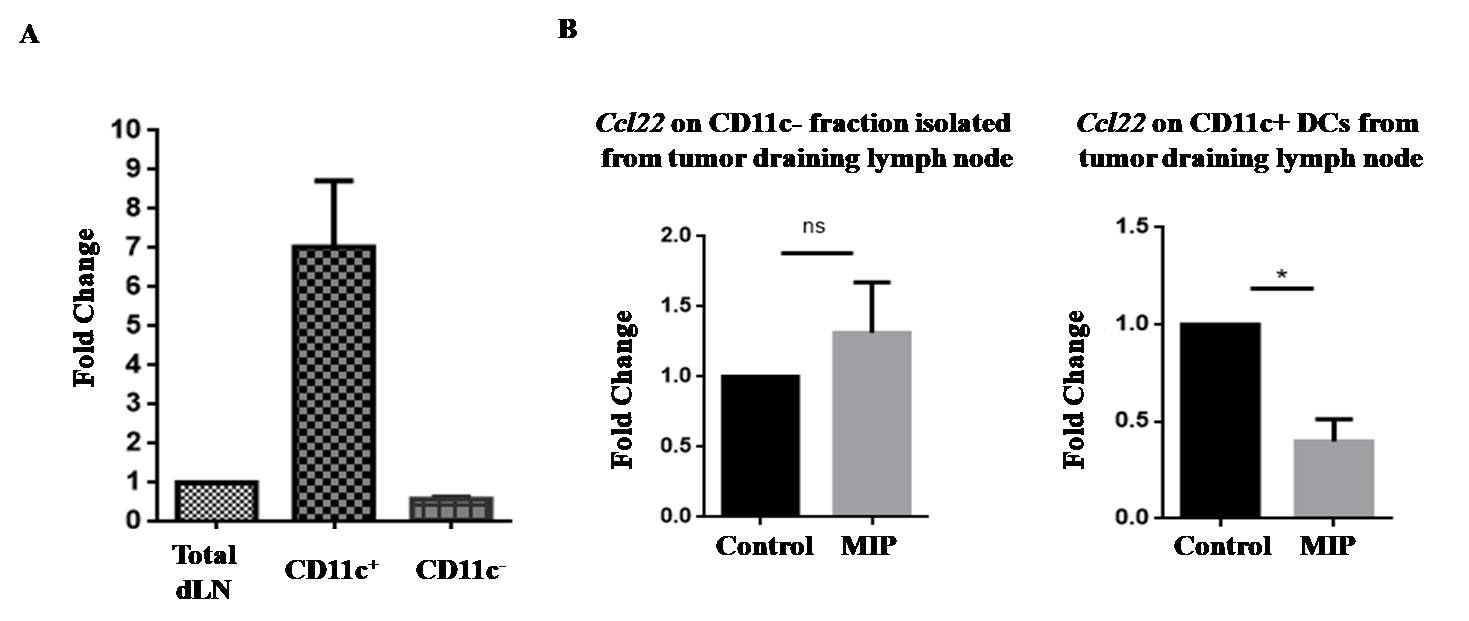


**Figure S4**. **Cellular source of *Ccl*22 in B16F10 TME: (A)** Single cell suspension of tumor draining lymph nodes was prepared (3 mice were pooled in each group). Using a pan-DC enrichment kit, CD11c^+^ DCs were enriched and *Ccl*22 mRNA levels were quantified by qRT-PCR in total leukocytes from tumor draining lymph node / CD11c^+^ DCs fraction / CD11c^-^ fraction. **(B)** Role of MIP immunotherapy in remodulation of *Ccl*22 expression on CD11c^+^ and CD11c^-^ fraction was evaluated. Results are represented as average of 5 mice per group. All results shown in bar graphs represent mean ± SEM. Statistical significance was determined by unpaired non-parametric student’s t-test (*: p<0.05, ns=non-significant). Two independent experimental repeats were done.


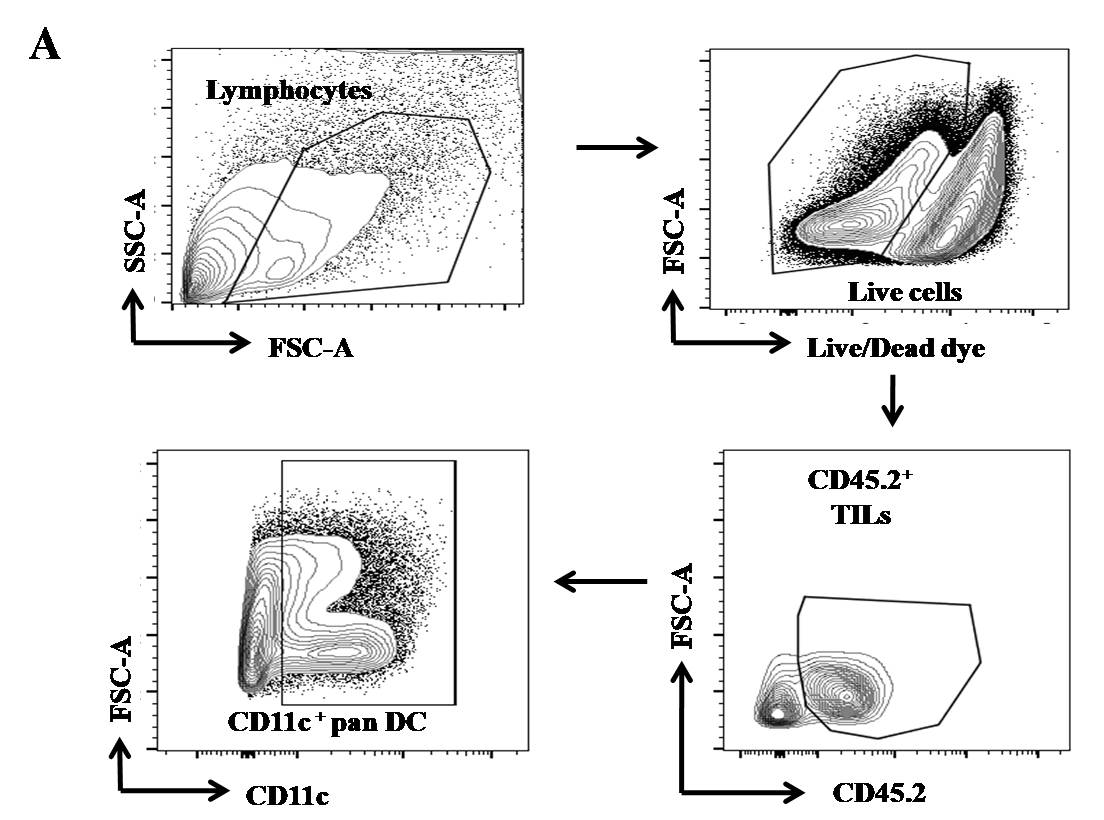


**
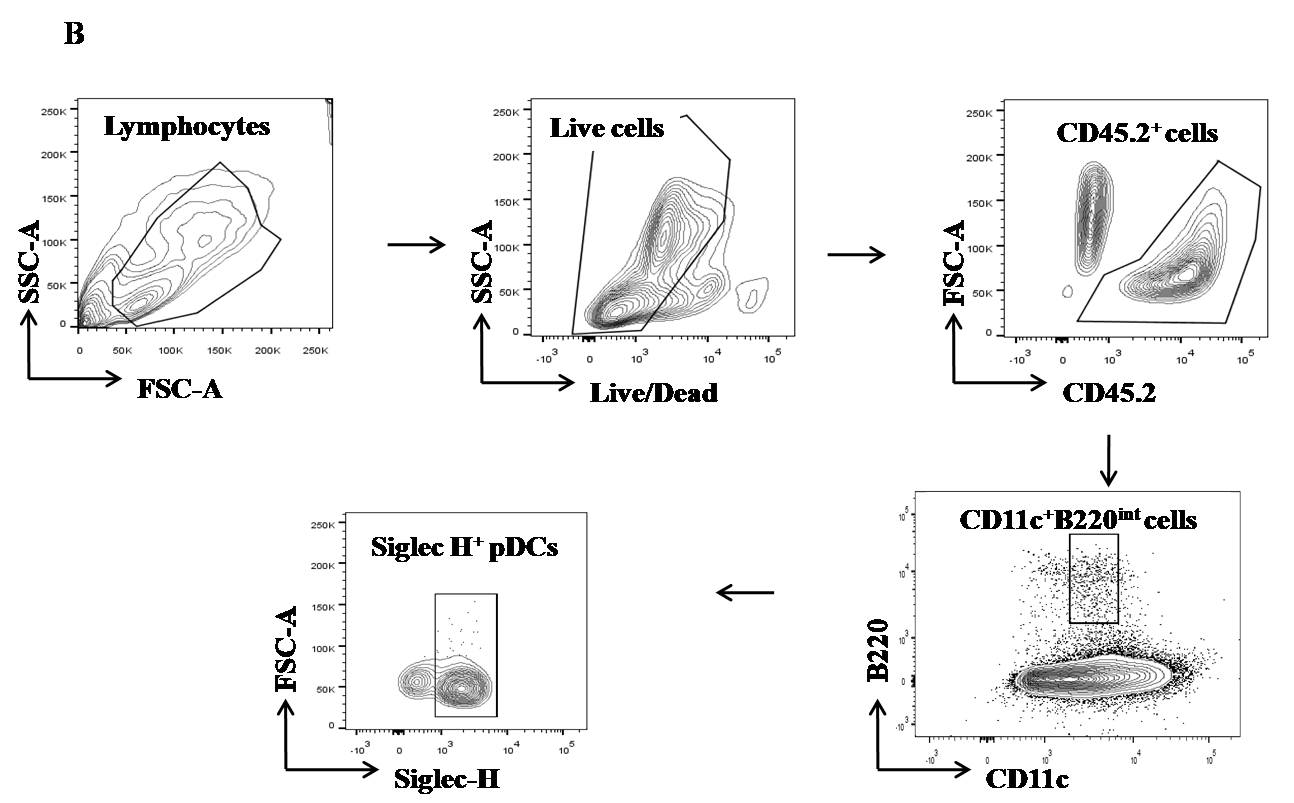
**

**Figure S5. (A) Gating strategy for analyzing tumor Ti-DCs:** For analyzing total Ti-DCs, lymphocytes were selected on the basis of size by plotting FSC-A against SSC-A followed by gating for live cells. Live cells were further sub-gated for tumor infiltrating leukocytes which were CD45.2positive. CD11c^+^ dendritic cells were selected from live CD45.2^+^ lymphocytes.  **(B) Gating strategy for analyzing tumor infiltrating Plasmacytoid DCs:** Lymphocytes were selected on the basis of size by plotting FSC-A against SSC-A followed by gating for live cells. Live cells were further sub-gated for tumor infiltrating leukocytes which were CD45.2positive. CD11c^+^B220^int^ dendritic cells were selected from live CD45.2^+^CD11c^+^DCs followed by gating for Siglec-H^+^ plasmacytoid DCs.


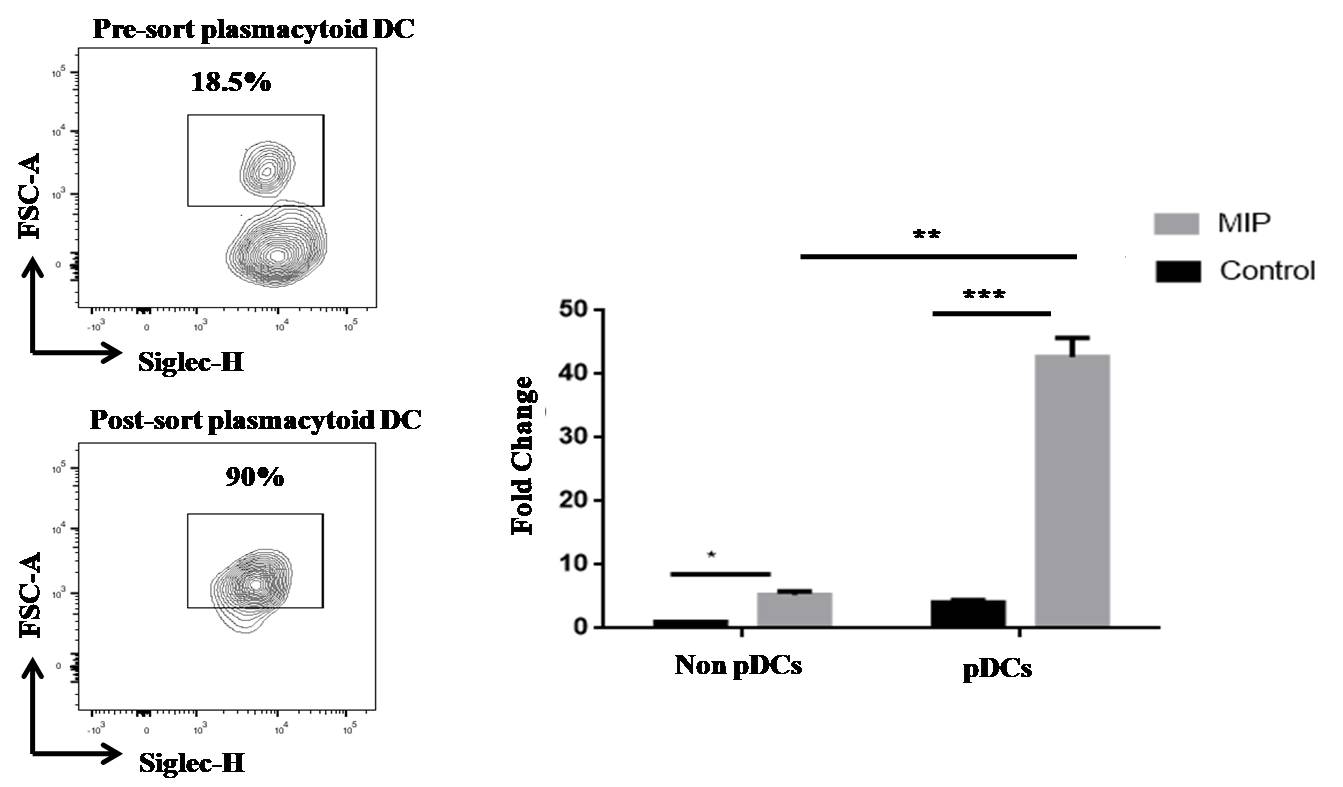


**Figure S6**. ***Ifn-α* expression in B16F10 TME of control and MIP treated mice. (A)** Single cell suspension of tumor draining lymph nodes were prepared (3 mice were pooled in each group). Using a plasmacytoid DC enrichment kit, pDCs were enriched and *Ifn-α* levels were quantified in pDCs fraction / non-pDC fraction. The contour plot represents the purity of pDCs after enrichment. **(B)** Role of MIP immunotherapy in remodulation of *Ifn-α* expression on pDC and non-pDC fraction was evaluated. Results are represented as average of 5 mice per group. All results shown in bar graphs represent mean ± SEM. Statistical significance was determined by 2-way ANOVA. Two independent experimental repeats were done.

**
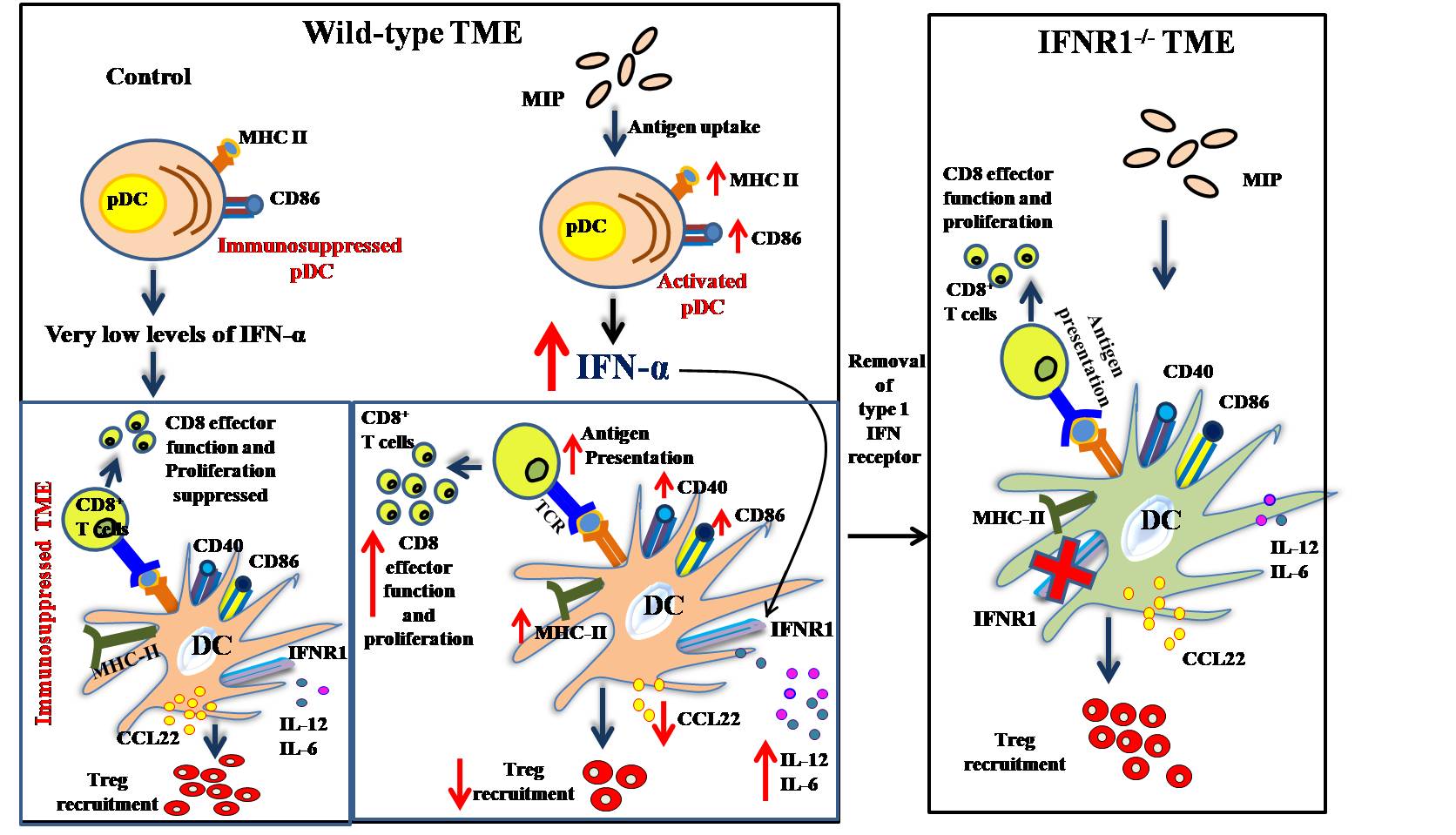
**

**Figure S7. A proposed model based on the observations of the current study:** It depicts the mechanism by which MIP immunotherapy increased the effector function of tumor infiltrating leukocytes to initiate efficient anti-tumor responses. In the control TME, Plasmacytoid DCs are functionally suppressed. Administration of MIP immunotherapy in the tumor activates pDCs which in turn increase the production of IFN-α. This pleiotropic cytokine actively modulates the immunosuppressed Ti-DCs towards pro-inflammatory phenotype as a result of which the secretion of CCL22 is strongly suppressed thereby leading to reduced frequency of immunosuppressive Tregs in TME. Apart from reducing Treg frequency, MIP treated Ti-DCs actively participate in increasing effector CD8^+^ T cell proliferation and function which ultimately results in effective control of tumor growth.

## Supplementary Tables

1. Table S1

| **Antibodies** | **Fluorochrome** | **Clone** | **Catalogue**  **No.** | **Make** |
| --- | --- | --- | --- | --- |
| **CD3** | **PECy7** | 17-A2 | 100219 | BioLegend |
| **CD4** | **VioBlue** | REA604 | 130-118-568 | Miltyni |
| **CD8** | **AF700** | 53-6.7 | 100730 | BioLegend |
| **CD45.2** | **PETR** | 104(RUO) | 565390 | BD |
| **Viability**  **dye** | **Amcyan** | - | 65-0866-18 | Invitrogen |
| **CD11c** | **VioBlue** | N418 | 565452 | BD |
| **SiglecH** | **FITC** | 551 | 129604 | BioLegend |
| **PDCA1** | **PECy7** | BST2 | 25-3172-82 | Invitrogen |
| **CD69** | **FITC** | H1.2F3 | 104506 | BioLegend |
| **Ki67** | **FITC** | SolA15 | 11-5698 | Invitrogen |
| **CD80** | **FITC** | 16-10A1 | 561954 | BD |
| **CD86** | **APC** | GK1 | 564198 | Invitrogen |
| **CD40** | **PE** | 1C10 | 12-0401-82 | Invitrogen |
| **MHC-II** | **APC** | M5/114.15.  2 | 17-5321-82 | Invitrogen |
| **IL-6** | **FITC** | MP5-20F3 | 11-7061-82 | Invitrogen |
| **IL-12** | **PerCPCy5.5** | C15.6 | 505211 | BioLegend |
| **IL-10** | **APC** | JES5-16E3 | 554468 | BD |
| **CTLA-4** | **PE** | UC10-4B9 | 106306 | BioLegend |
| **PD-1** | **BV421** | 29F.1A12 | 135221 | BioLegend |
| **B220** | **PECy7** | RA3-6B22 | 103222 | BioLegend |
| **CD3** | **FITC** | 17-A2 | 100204 | BioLegend |
| **B220** | **APC** | RA3-6B22 | 103212 | BioLegend |
| **IL-10** | **AF700** | JES5-16E3) | 56-7101-82 | Invitrogen |

1. Table S2

| **Gene** | **Primersequences(5’-3’)** |
| --- | --- |
| *Ifn-α*(F) | GGACTTTGGATTCCCGCAGGAGAAG |
| *Ifn-α*(R) | GCTGCATCAGACAGCCTTGCAGGTC |
| *Ccl4* (F) | AAACCTAACCCCGAGCAACA |
| *Ccl4* (R) | CCATTGGTGCTGAGAACCCT |
| *Ccl5*(F*)* | GTGCTCCAATCTTGCAGTCG |
| *Ccl5* (R) | AGAGCAAGCAATGACAGGGAA |
| *Ccl22*(F) | CAAGCCTGGCGTTGTTTTGAT |
| *Ccl22*(R) | GCAAGGCTCTTGCTGGAATG |
| *Ccr5* (F) | GACATCCGTTCCCCCTACAAG |
| *Ccr5*(R) | TCACGCTCTTCAGCTTTTTGCAG |
| *Ifn-β*(F) | CTCCACCAGCAGACAGTGTT |
| *Ifn-β*(R) | CCAGGCGTAGCTGTTGTACT |
| *Cxcr3*(F) | CAAGCCATGTACCTTGAGGTTAG |
| *Cxcr3*(R) | TCAGGCTGAAATCCTGTGGG |
| *Ccl17*(F) | ACTTCAAAGGGGCCATTCCT |
| *Ccl17*(R) | TGGACAGTCAGAAACACGATG |
| *Ccr4(F)* | CTTTCAGAAGAGCAAGGCAGCTC |
| *Ccr4(R)* | GGCTTTGGCATGCTTTCGTA |
| *Ccr7(F)* | CATGGACCCAGGTGTGCTT |
| *Ccr7(R)* | CATGAGAGGCAGGAACCAGG |
| *Ccxl10(F)* | CCACGTGTTGAGATCATTGCC |
| *Ccxl10(F)* | GAGGCTCTCTGCTGTCCATC |
